# Examining the impacts of DM filters to PFC isolated Ćuk converter for DCM operation by comparing Si and SiC MOSFET

**DOI:** 10.1038/s41598-023-31965-2

**Published:** 2023-03-23

**Authors:** Erdal Şehirli

**Affiliations:** Electrical-Electronics Engineering, Kastamonu University, 37150 Kastamonu, Turkey

**Keywords:** Electrical and electronic engineering, Engineering

## Abstract

Examining impacts of differential mode (DM) filter topologies covering pi, LC with damping, LC, LCL filter through isolated Ćuk single phase PFC converter is realized in this paper regarding to power factor, THD and efficiency. Application of PFC isolated Ćuk converter is conducted with 42 kHz switching frequency and 50 W power. Each filter is modeled, designed, and applied experimentally via isolated PFC Ćuk converter. Average model derivation based on state-space, for DCM operation of input side inductor of isolated PFC Ćuk converter that is not introduced in literature is the main contribution of the paper. Second main contribution is to analyze filter types by linear methods for the transfer functions cascading the converter and filter transfer functions, which is not presented for PFC converters. It is also presented that solely LCL filter which is not analyzed for PFC converters in detail in literature, does not give desired results. So, increasing the effectivity of LCL filter, new filter structure LCL with parallel C filter is proposed and modeled in this paper. Thanks to the applications, presented results of LCL with parallel C filter is better than others with 4.9% current THD and ‘1’ power factor. Proposed LCL filter ensures 45% reduction of total inductor value, comparing to LC filter. Besides, LCL with C filter provides better control characteristics with maximum allowable gain for stability as 0.7585 higher than other topologies. Moreover, practical design methodology of filter types avoiding complex mathematical procedure is given in this paper. Applications with each filters provide THD requirements, obtained 4.9% as a best value, lower than IEC61000-3-2 standard. Maximum percentage improvement comparing to the IEC61000-3-2 is 27.5% for third harmonic. Furthermore, SiC and Si Mosfets are employed separately in the converter and compared by using each DM filters regarding to power factor, THD, efficiency through applications. As a result, better efficiency with SiC Mosfet as 83% and better THD with Si Mosfet as 4.9% are conducted.

## Introduction

Design of filter named as electromagnetic interference (EMI) is very important part of the power electronics because of decreasing the noise that are common and differential mode noises (CM), (DM), respectively. Operation of the converter is not achieved as it desired and efficiency, power factor, THD may be reduced, if the DM filter is not designed appropriately. DM filter design has great importance especially for PFC AC-DC converters due to providing lower THD, higher power factor (PF) and continuous input current for DCM operation of the converter. Although different kind of converter topologies for PFC purpose can be used, in this paper isolated Ćuk converter as a PFC converter is chosen because of the need for isolated converter topologies notedly for battery charging purposes. One of the main reasons of choosing isolated Ćuk converter in this paper is having two inductors, one is at input side, the other is at output side, providing less ripple current for both input and output of the converter. It also provides isolation. On the other hand, other topologies such as isolated SEPIC and isolated Zeta have just one inductor at input side or at output side, respectively that may result higher current ripple at the side having no inductor. Also, another advantage of isolated Ćuk converter is having just one power switch comparing to totem pole or dual active bridge converters. Besides, isolated Ćuk converter have a low side switch that ensures easiness to control power switch comparing to Zeta, totem pole or dual active bridge converters. Moreover, comparison of wide bandgap semiconductors with respect to THD and PF for PFC converters by using DM filters is not presented in detail even though application of wide bandgap semiconductor in converters has becoming very popular.

Some studies in literature are listed as follows in terms of filter types, design and application of the filter types, analysis of the filter regarding to control point of view, comparison of the filter types, the use of wide bandgap semiconductor with converter through DM filter. EMI filter types used in the literature are given with the emphasis of DM noise reduction in^1^.

Although, fundamental design and application of LC DM filter is conducted in^2^ for PFC converter, in^3^ for DC–DC converter, in^4^ for inverter, comparison, modeling and analyzing regarding to control point of view is not presented. Application and design of LC with damping DM filter is also conducted in^5^ for inverter, in^6^ for DC–DC converters without analyzing in terms of control point of view. Similarly, pi DM filter is applied for PFC converter in^7^, without modeling and analysis. Even tough, LCL DM filter is applied for three phase inverter in^8–12^ and for three phase rectifiers in^13–16^, application, design and comparison of LCL DM filter for single phase PFC converter is not conducted.

Analysis of the filter types regarding to the control characteristics using linear control techniques like root locus is realized for three phase inverters in^17,18^ for three phase rectifiers in^19^, for DC–DC converters in^20^ by using LCL filter, without comparison except^20^. Similarly, analysis and modeling of LC filter is conducted for three phase inverters in^21–23^, for three phase rectifiers in^24–26^ and for DC–DC converter in^27–29^ without comparison of filter types regarding modeling and control characteristics except in^20^. None of the mentioned studies includes analysis of the DM filter types for single phase PFC regarding to control point of view.

Although comparison of LCL, LC and L filters are conducted for three phase inverters in^30,31^, for three phase rectifiers in^32^, for DC–DC converter in^20^, analysis of control characteristics by linear techniques are not included except^20^. In addition^33,34^, compare L, LC and LCL filter through single phase inverter with respect to control point of view, however it does not include comparison of PF and THD.

Besides, the use of SiC wide band gap semiconductor with LCL filter is conducted for three phase inverters in^35,36^, for three phase rectifiers in^37,38^, for single phase PFC boost converter in^39^, for singe phase PWM rectifier in^40^, however comparison regarding to THD, efficiency and PF is not presented.

As a result of literature survey of EMI DM filter, for single phase PFC converter, analysis, design, and comparison is not presented in literature. Therefore, design, modeling, analysis, and transfer functions of DM filter topologies consisting of pi, LC, LC with damping, LCL filters is presented in this paper. Also, each of the DM filters are applied by PFC isolated Ćuk converter operated at DCM up to 50 W power and comparisons are made. Furthermore, small signal analysis and average state space model derivation for DCM operation of input side inductor of isolated PFC Ćuk converter which is not introduced in literature are conducted. Analyzing cascaded transfer functions of DM filters and the converter through bode and root locus which are linear control techniques, for PFC converter is unique contribution of the study. Owing to the experiments, it is realized that just LCL filter does not provide desired results. So, in this paper, new DM filter structure consisting of LCL with parallel C filter is presented. It is proved that proposed LCL with parallel C filter ensures better outcomes than pi, LC with damping, LC by giving 4.9% current THD and ‘1’ PF. Also, it ensures 45% total inductor reduction regarding to LC filter. Besides, LCL with C filter has the better control characteristics by providing maximum allowable gain for stability as 0.7585. Furthermore, all DM filters designed and applied for single phase PFC isolated Ćuk converter provide the limits stated in IEC61000-3-2 standards, obtained 4.9% as a best value. Maximum percentage improvement comparing to the IEC61000-3-2 is 27.5% for third harmonic. Each application for all DM filter topologies is conducted with Si and SiC Mosfet independently, and exact comparisons are made by considering efficiency, THD and PF. Although the use of wide bandgap semiconductor has recently gained much attention, exact comparison for DM filter with PFC considering efficiency, THD and PF are not presented in detail in literature. However, this paper presents such comparison as a contribution to the literature. As a result, better efficiency with SiC Mosfet as 83% and better THD with Si Mosfet as 4.9% are acquired.

Main contributions of the paper to the literature can be listed as follows.

- DCM model including transfer function and small-signal analysis of PFC isolated AC-DC Ćuk converter is derived.
- DM filter topologies covering LC, LC with damping, pi and LCL, are compared regarding to control point of view, THD, PF and efficiency through PFC isolated AC-DC Ćuk converter with applications. Besides, cascaded transfer functions including each filter type is derived considering the converter.
- It is also shown that for PFC converter, traditional LCL DM filter is not suitable to increase PF and reduce THD. New LCL type DM filter is presented to have better result.
- Comparison of Si and SiC Mosfet is conducted by using each DM filter structures for PFC converter.

## Isolated ĆUK PFC AC-DC converter based on DCM

**Figure 1:**
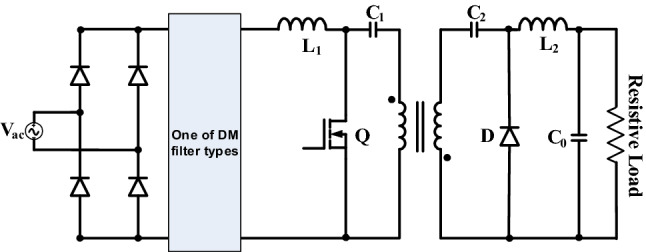
PFC single phase isolated Ćuk AC-DC converter.

Figure 1 depicts AC-DC isolated PFC Ćuk single phase converter structure. In addition, single phase grid connection is provided by diode bridge through DM filter.

**Figure 2:**
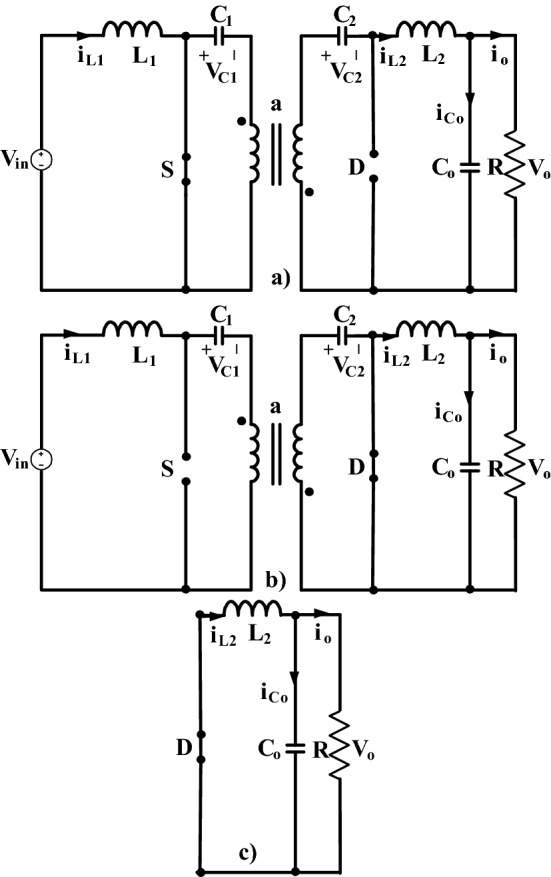
(**a**) Switch on equivalent circuit (**b**,**c**) switch off equivalent circuits of isolated PFC Ćuk converter.

Working principle of the converter can be defined as follows by Fig. 2; L_1_ is energized and stores energy, C_1_ discharges to secondary winding of transformer through primary windings, C_2_ discharges to L_2_ and to load while the switch is on. At the first switch off duration, L_1_ and input source charge C_1_, and C_2_ by diode, also L_2_ feeds the load. At the second switch off duration, L_2_ feeds the load by power diode. Parameters of the converter is chosen by using (1), (2), (3), (4), (5). In the applications, L_1_ is chosen for DCM operating mode as 1180 μH and L_2_ is chosen for CCM operating mode as 654 μF.

1 $${{L}}_{1}  = \frac{{{R}}_{{L}}{{(1-D)}}^{2}}{{{2D}}{{f}}_{{s}}{{{n}}}^{2}} = \frac{{12.5}{(1-0.45)}^{2}}{{2  \times  0.45  \times  42000}{0.2}^{2}}{=2.5} \; \text{mH}$$

2 $${{L}}_{2} = \frac{{{R}}_{{L}}{(1-D)}}{{2}{{f}}_{{s}}} = \frac{12.5(1-0.45)}{{2  \times  42000}}{=81.85} \; \upmu\text{H}$$

3 $${{C}}_{1} = \frac{{{V}}_{{in}}{{{n}}}^{2}{{{D}}}^{2}}{{(1-D)\Delta}{{V}}_{{C1}}{{{f}}}_{{s}}{{{R}}}_{{L}}} = \frac{{{142}  \times  }{0.2}^{2}{{0.45}}^{2}}{{(1-0.45) \times  10 \times 42000  \times  12.5}} = {0.39} \; \upmu\text{F}$$

4 $${{C}}_{2} = \frac{{{V}}_{{o}}{{D}}}{{\Delta}{{V}}_{{C2}}{{{f}}}_{{s}}{{{R}}}_{\text{L}}} = \frac{{25  \times  0.45}}{{22  \times  42000  \times  12.5}} {=0.97} \; \mu\text{F}$$

5 $${{C}}_{0} \ge \frac{{{V}}_{{o}}{(1-D)}}{{8}{{L}}_{2}{\Delta}{{V}}_{{C0}}{{{f}}}_{{s}}^{2}} = \frac{25(1-0.45)}{{{8  \times  654  \times  1 \times  }}{42000}^{2}{ \times }{10}^{-6}}{=1.48 } \; \upmu\text{F}$$

To examine the impact of DM filters to the PFC isolated Ćuk converter, firstly converter’s mathematical model must be obtained. For DCM operation, average state space model derivation of isolated Ćuk PFC converter is one of the unique novelties of the study. Although, average circuit model of Ćuk isolated DC-DC converter is derived in^41^, state space average model especially for DCM operation of input side inductor is not presented in literature. This study aims converter’s DCM operation because in DCM operation PFC can be provided just by output voltage regulation without the need of controlling input side inductor current. Average state space model derivation of DCM operation for isolated PFC Ćuk converter includes three intervals considering switch on and off states. The modes of operation are shown in Fig. 2. Mode one is when S is on, mode two and three are when S is off. The model is derived by applying Kirchhoff Current and Voltage Law to each interval. In Fig. 2, a is the turn ratio of high frequency transformer.

At the switch on interval in Fig. 2a, after applying Kirchhoff Voltage and Current Laws, state equations given in (6), (7), (8), (9), (10) are obtained. It is seen that there are five state variables as i_L1_, i_L2_, v_C1_, v_C2_, v_o_^20^.

6 $$\frac{{{di}}_{{L1}}}{{dt}} = \frac{{{V}}_{{in}}}{{{L}}_{1}}$$

7 $$\frac{{{dV}}_{{C1}}}{{dt}} = \frac{{{-ai}}_{{L2}}}{{{C}}_{1}}$$

8 $$\frac{{{di}}_{{L2}}}{{dt}} = \frac{{{V}}_{{C1}}}{{{a}}{{L}}_{2}}{+}\frac{{{V}}_{{C2}}}{{{L}}_{2}}-\frac{{{V}}_{0}}{{{L}}_{2}}$$

9 $$\frac{{{dV}}_{{C2}}}{{dt}} = \frac{{{i}}_{{L2}}}{{{C}}_{2}}$$

10 $$\frac{{{dV}}_{0}}{{dt}} = \frac{{{i}}_{{L2}}}{{{C}}_{0}}-\frac{{{V}}_{0}}{{{RC}}_{0}}$$

After writing state equations in (6), (7), (8), (9), (10) with the form of (11), mathematical model for switch on interval is obtained in (12).

11 $$\dot{{x}}{=Ax+Bu}$$

12 $$\dot{\left[\begin{array}{c}{{i}}_{{L1}}\\ {{V}}_{{C1}}\\ {{i}}_{{L2}}\\ {{V}}_{{C2}}\\ {{V}}_{0}\end{array}\right]}{ = }\left[\begin{array}{ccccc}{0}& {0}& {0}& {0}& {0}\\ {0}& {0}& \frac{{-a}}{{{C}}_{1}}& {0}& {0}\\ {0}& \frac{1}{{{a}}{{L}}_{2}}& {0}& \frac{1}{{{L}}_{2}}& \frac{-1}{{{L}}_{2}}\\ {0}& {0}& \frac{1}{{{C}}_{2}}& {0}& {0}\\ {0}& {0}& \frac{1}{{{C}}_{0}}& {0}& \frac{-1}{{{R}}{{C}}_{0}}\end{array}\right]\left[\begin{array}{c}{{i}}_{{L1}}\\ {{V}}_{{C1}}\\ {{i}}_{{L2}}\\ {{V}}_{{C2}}\\ {{V}}_{0}\end{array}\right]{+}\left[\begin{array}{c}\frac{1}{{{L}}_{1}}\\ {0}\\ {0}\\ {0}\\ {0}\end{array}\right]{{V}}_{{in}}$$

In DCM operation, there are two switch-off intervals. For first switch off interval in Fig. 2b, state equations are obtained as in (13), (14), (15), (16), (17), after applying Kirchhoff Current and Voltage Laws.

13 $$\frac{{{di}}_{{L1}}}{{dt}} = \frac{{{-V}}_{{C1}}}{{{L}}_{1}}-\frac{{{a}}{{V}}_{{C2}}}{{{L}}_{1}}{+}\frac{{{V}}_{{in}}}{{{L}}_{1}}$$

14 $$\frac{{{dV}}_{{C1}}}{{dt}} = \frac{{{i}}_{{L1}}}{{{C}}_{1}}$$

15 $$\frac{{{di}}_{{L2}}}{{dt}}{=-}\frac{{{V}}_{0}}{{{L}}_{2}}$$

16 $$\frac{{\text{dV}}_{{C2}}}{{dt}} = \frac{{{a}}{{i}}_{{L1}}}{{{C}}_{2}}$$

17 $$\frac{{{dV}}_{0}}{{dt}} = \frac{{{i}}_{{L2}}}{{{C}}_{0}}-\frac{{{V}}_{0}}{{{RC}}_{0}}$$

State equations is written with the form of (11) to have mathematical model of first switch off interval in (18).

18 $$\dot{\left[\begin{array}{c}{{i}}_{{L1}}\\ {{V}}_{{C1}}\\ {{i}}_{{L2}}\\ {{V}}_{{C2}}\\ {{V}}_{0}\end{array}\right]}{ = }\left[\begin{array}{ccccc}{0}& \frac{-1}{{{L}}_{1}}& {0}& \frac{{-a}}{{{L}}_{1}}& {0}\\ \frac{1}{{{C}}_{1}}& {0}& {0}& {0}& {0}\\ {0}& {0}& {0}& {0}& \frac{-1}{{{L}}_{2}}\\ \frac{{a}}{{{C}}_{2}}& {0}& {0}& {0}& {0}\\ {0}& {0}& \frac{1}{{{C}}_{0}}& {0}& \frac{-1}{{{R}}{{C}}_{0}}\end{array}\right]\left[\begin{array}{c}{{i}}_{{L1}}\\ {{V}}_{{C1}}\\ {{i}}_{{L2}}\\ {{V}}_{{C2}}\\ {{V}}_{0}\end{array}\right]{+}\left[\begin{array}{c}\frac{1}{{{L}}_{1}}\\ {0}\\ {0}\\ {0}\\ {0}\end{array}\right]{{V}}_{{in}}$$

Second switch off interval in Fig. 2c is the interval that the current of i_L1_ stays at ‘0’. Assuming the condition of (19), state equations of second switch off interval is obtained as in (20), (21).

19 $${{i}}_{{L1}}{=0, }{{V}}_{{C1}} = {{V}}_{{C2}} = \frac{{{di}}_{{L1}}}{{dt}}= {0} $$

20 $$\frac{{{di}}_{{L2}}}{{dt}}{=-}\frac{{{V}}_{0}}{{{L}}_{2}}$$

21 $$\frac{{{dV}}_{0}}{{dt}} = \frac{{{i}}_{{L2}}}{{{C}}_{0}}-\frac{{{V}}_{0}}{{{RC}}_{0}}$$

After writing second switch off interval with the form of (11), (22) is obtained.

22 $$\dot{\left[\begin{array}{c}{{i}}_{{L1}}\\ {{V}}_{{C1}}\\ {{i}}_{{L2}}\\ {{V}}_{{C2}}\\ {{V}}_{0}\end{array}\right]}{ = }\left[\begin{array}{ccccc}{0}& {0}& {0}& {0}& {0}\\ {0}& {0}& {0}& {0}& {0}\\ {0}& {0}& {0}& {0}& \frac{-1}{{{L}}_{2}}\\ {0}& {0}& {0}& {0}& {0}\\ {0}& {0}& \frac{1}{{{C}}_{0}}& {0}& \frac{-1}{{{R}}{{C}}_{0}}\end{array}\right]\left[\begin{array}{c}{{i}}_{{L1}}\\ {{V}}_{{C1}}\\ {{i}}_{{L2}}\\ {{V}}_{{C2}}\\ {{V}}_{0}\end{array}\right]{+}\left[\begin{array}{c}\frac{1}{{{L}}_{1}}\\ {0}\\ {0}\\ {0}\\ {0}\end{array}\right]{{V}}_{{in}}$$

For deriving state-space representation of the PFC isolated Ćuk converter given in (25), state-space model of each three-switching intervals is written with the form of (23), (24).

23 $${A} = {{dA}}_{1}+\delta{{A}}_{2}{+}{{(1-\delta-d)A}}_{3}$$

24 $${B} = {{dB}}_{1}{+\delta}{{B}}_{2}{+}{{(1-\delta-d)B}}_{3}$$

25 $$\dot{\left[\begin{array}{c}{{i}}_{{L1}}\\ {{V}}_{{C1}}\\ {{i}}_{{L2}}\\ {{V}}_{{C2}}\\ {{V}}_{0}\end{array}\right]}{ = }\left[\begin{array}{ccccc}{0}& \frac{-\delta}{{{L}}_{1}}& {0}& \frac{{-a\delta}}{{{L}}_{1}}& {0}\\ \frac{\delta}{{{C}}_{1}}& {0}& \frac{{-da}}{{{C}}_{1}}& {0}& {0}\\ {0}& \frac{{d}}{{{aL}}_{2}}& {0}& \frac{{d}}{{{L}}_{2}}& \frac{-1}{{{L}}_{2}}\\ \frac{{a\delta}}{{{C}}_{2}}& {0}& {0}& {0}& {0}\\ {0}& {0}& \frac{1}{{{C}}_{0}}& {0}& \frac{-1}{{{R}}{{C}}_{0}}\end{array}\right]\left[\begin{array}{c}{{i}}_{{L1}}\\ {{V}}_{{C1}}\\ {{i}}_{{L2}}\\ {{V}}_{{C2}}\\ {{V}}_{0}\end{array}\right]{+}\left[\begin{array}{c}\frac{1}{{{L}}_{1}}\\ {0}\\ {0}\\ {0}\\ {0}\end{array}\right]{{V}}_{{in}}$$

To present the effect of the DM filter topologies on PFC isolated Ćuk converter, converter’s transfer function should be derived. To achieve small signal model, (26) must be applied to the isolated PFC Ćuk converters’ state space model. In (26), k is equal to $\delta$/d.

26 $$\dot{\widetilde{{x}}}{=(}{{dA}}_{1}{+\delta}{{A}}_{2}{+}{\left({1-kd-d}\right){{A}}}_{3}{)}\widetilde{{x}}{+[(}{{A}}_{1}+ {k} {{A}}_{2}-{\left({k+1}\right){{A}}}_{3}{)}\stackrel{\mathrm{-}}{{x}}{+(}{{B}}_{1}+ {k} {{B}}_{2}-{\left({k+1}\right){{B}}}_{3}{)u]}$$

After applying (26) to (12), (18), (22), small signal model is obtained in (27).

27 $$\dot{\left[\begin{array}{c}{{i}}_{{L1}}\\ {{V}}_{{C1}}\\ {{i}}_{{L2}}\\ {{V}}_{{C2}}\\ {{V}}_{0}\end{array}\right]}{ = }\left[\begin{array}{ccccc}{0}& \frac{{-kd}}{{{L}}_{1}}& {0}& \frac{{-akd}}{{{L}}_{1}}& {0}\\ \frac{{kd}}{{{C}}_{1}}& {0}& \frac{{-da}}{{{C}}_{1}}& {0}& {0}\\ {0}& \frac{{d}}{{{aL}}_{2}}& {0}& \frac{{d}}{{{L}}_{2}}& \frac{-1}{{{L}}_{2}}\\ \frac{{akd}}{{{C}}_{2}}& {0}& \frac{{d}}{{{C}}_{2}}& {0}& {0}\\ {0}& {0}& \frac{1}{{{C}}_{0}}& {0}& \frac{-1}{{{R}}{{C}}_{0}}\end{array}\right]\widetilde{\left[\begin{array}{c}{{i}}_{{L1}}\\ {{V}}_{{C1}}\\ {{i}}_{{L2}}\\ {{V}}_{{C2}}\\ {{V}}_{0}\end{array}\right]}{+}\left[\begin{array}{c}\frac{-{{V}}_{{C1}}{k} - {{V}}_{{C2}}{{ka}}}{{{L}}_{1}}{+}\frac{{(1+k)}{{V}}_{{in}}}{{{L}}_{1}}\\ \frac{{{k}}{{i}}_{{L1}}{-a}{{i}}_{{L2}}}{{{C}}_{1}}\\ \frac{{{V}}_{{C1}}+ {a} {{V}}_{{C2}}}{{{aL}}_{2}}\\ \frac{{{ak}}{{i}}_{{L1}}+ {a} {{i}}_{{L2}}}{{{C}}_{2}}\\ {0}\end{array}\right]\widetilde{{d}}$$

For isolated PFC Ćuk converter, transfer function of control to output voltage v_o_ is derived in (28) by applying C(sI-A)^−1^B equality to (27).

28 $$T\left(s\right)=\frac{\widetilde{{V}_{0}}}{\widetilde{d}}=\frac{\begin{array}{c}{s}^{3}\frac{\left({V}_{in}-\frac{{V}_{in}d}{d-1}\right)}{{C}_{0}{L}_{2}a}+{s}^{2}\left(\frac{d\left(\frac{{V}_{in}k{d}^{2}}{R{\left(d-1\right)}^{2}} - \frac{{dV}_{in}}{Ra\left(d-1\right)}\right)}{{C}_{0}{L}_{2}{C}_{2}}+ \frac{d\left(\frac{{V}_{in}k{d}^{2}}{R{a\left(d-1\right)}^{2}}+ \frac{{dV}_{in}}{R\left(d-1\right)}\right)}{{C}_{0}{L}_{2}{C}_{1}a}\right)+\\ s\left(\begin{array}{c}\frac{{d}^{2}{k}^{2}\left({V}_{in}-\frac{{V}_{in}d}{d-1}\right)}{{C}_{0}{L}_{2}{C}_{1}{L}_{1}a}- \frac{{d}^{2}{k}^{2}\left(\frac{\left({V}_{in}k-\frac{{V}_{in}d}{d-1}\right)}{{L}_{1}} - \frac{{\left(1+k\right)V}_{in}}{{L}_{1}}\right)}{{C}_{0}{L}_{2}{C}_{1}a} -\\ \frac{{d}^{2}ka\left(\frac{\left({V}_{in}k - \frac{{V}_{in}d}{d-1}\right)}{{L}_{1}} - \frac{{\left(1+k\right)V}_{in}}{{L}_{1}}\right)}{{C}_{0}{L}_{2}{C}_{2}}+\frac{{d}^{2}{k}^{2}a\left({V}_{in}-\frac{{V}_{in}d}{d-1}\right)}{{C}_{0}{L}_{2}{C}_{1}{L}_{1}}\end{array}\right)\end{array}}{\begin{array}{c}{s}^{5}+\frac{{s}^{4}}{R{C}_{0}}+{s}^{3}\left(\frac{{d}^{2}}{{C}_{0}{L}_{2}} +\frac{{-d}^{2}}{{C}_{1}{L}_{2}} + \frac{{d}^{2}{k}^{2}}{{C}_{1}{L}_{1}}+\frac{{d}^{2}{k}^{2}{a}^{2}}{{C}_{2}{L}_{1}}\right)+\\ {s}^{2}\left(\frac{{d}^{2}}{{C}_{1}{C}_{0}{L}_{2}R}-\frac{{d}^{2}}{{C}_{2}{C}_{0}{L}_{2}R}+\frac{{d}^{2}{k}^{2}}{{C}_{1}{C}_{0}{L}_{1}R}+\frac{{d}^{2}{k}^{2}{a}^{2}}{{C}_{1}{C}_{0}{L}_{1}R}\right)+\\ s\left(\frac{{d}^{2}{k}^{2}{a}^{2}}{{C}_{2}{C}_{0}{L}_{1}{L}_{2}}+\frac{{d}^{2}{k}^{2}}{{C}_{2}{C}_{0}{L}_{1}{L}_{2}}\right)\end{array}}$$

## DM filter types modeling and design

Isolated PFC Ćuk converters’ grid connection is realized by DM filter though diode bridge. As a DM filter, pi, LC, LCL, LC with damping filters are employed. Design methodology, transfer functions and mathematical models of each filter are introduced in this chapter.

### LC DM filter

**Figure 3:**
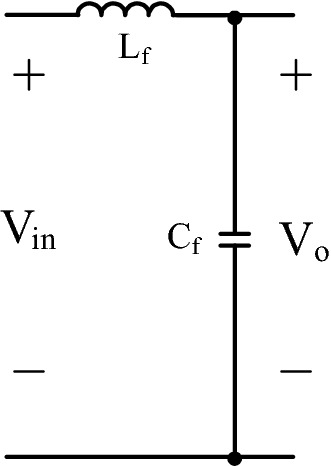
LC DM filter.

In Fig. 3, LC filter having a capacitor and inductor is shown. As a DM filter of PF converter, LC filter is generally used as in^2^.

Passive values of the LC filter can be calculated by using, (29), (30) as in^2^. (29) gives the maximum capacitor value, but filter capacitor value must be much lower than maximum value.

29 $${{C}}_{{fmax}}{=}\frac{{{P}}_{{max}}}{{\omega}_{{L}}{{{V}}}_{{in}}^{2}} {tan \theta, } \; \; {{C}}_{{fmax}}{\ll}{{C}}_{{f}}{, \;\; \theta=1}$$

30 $${{L}}_{{f}} = \frac{1}{{4}{{\pi}}^{2}{{{f}}}_{{c}}^{2}{{{C}}}_{{f}}}{, }{{f}}_{{sw}}{\ll}{{f}}_{{c}}$$

Filter inductor value can be calculated by using (30). Also, corner frequency (f_c_) must be approximately by ten times lower than (f_sw_) switching frequency, as in^2^. Mathematical model is derived in (31) after applying Kirchhoff voltage and current laws. Also, (32) gives filter transfer function.

31 $$\dot{\left[\begin{array}{c}{{i}}_{{{L}}_{{f}}}\\ {{V}}_{0}\end{array}\right]}{=}\left[\begin{array}{cc}{0}& -\frac{1}{{{L}}_{{f}}}\\ \frac{1}{{{C}}_{{f}}}& {0}\end{array}\right]\left[\begin{array}{c}{{i}}_{{{L}}_{{f}}}\\ {{V}}_{0}\end{array}\right] + \left[\begin{array}{c}\frac{1}{{{L}}_{{f}}}\\ {0}\end{array}\right]{{V}}_{{in}}$$

32 $${{T}}\left({{s}}\right) = \frac{{{V}}_{{o}}}{{{V}}_{{in}}} = \frac{1}{{{s}}^{2}{{{L}}}_{{f}}{{{C}}}_{{f}}+ {1} }$$

### LC DM filter with parallel damping

**Figure 4:**
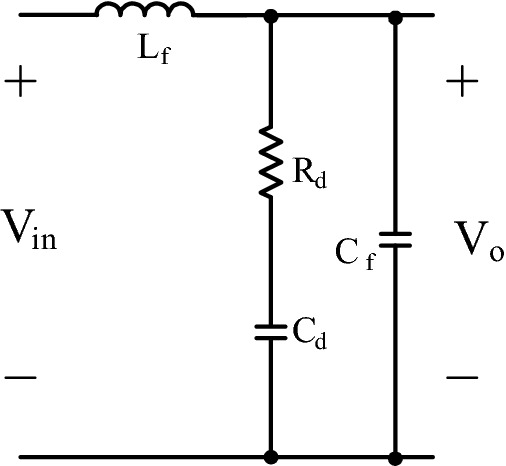
LC with damping DM filter.

Figure 4 gives LC with parallel damping filter. LC with parallel damping filter is mostly preferred in DC-DC converter as in^3,6^. The filter consists of series connected C_d_ and R_d_ that is added to LC filter to ensure damping.

Filter capacitor C_f_ and filter inductor L_f_ can be designed by using (29), (30). To calculate damping capacitor C_d_ and resistor R_d_, (33), (34), (35) are used as in^3,6^.

33 $${{R}}_{{in}} = \frac{{{{U}}_{{in}}}^{2}}{{{P}}_{{o}}}$$

34 $$\frac{{{L}}_{{f}}}{{{C}}_{{f}}{{{R}}}_{{d}}}{\ll}{{R}}_{{in}}$$

35 $${\zeta=}\frac{{n+1}}{{n}}\frac{{{L}}_{{f}}}{{2}{{R}}_{{d}}\sqrt{{{L}}_{{f}}{{{C}}}_{{f}}}}{, n=}\frac{{{C}}_{{d}}}{{{C}}_{{f}}}$$

n constant in (35) is generally taken as 4 as in^3^. Mathematical model in state space form is obtained in (36) after applying Kirchhoff voltage and current laws. Also, (37) gives filter’s transfer function.

36 $$\dot{\left[\begin{array}{c}{{i}}_{{{L}}_{{f}}}\\ {{V}}_{{{C}}_{{d}}}\\ {{V}}_{0}\end{array}\right]}{=}\left[\begin{array}{ccc}{0}& {0}& -\frac{1}{{{L}}_{{f}}}\\ {0}& -\frac{1}{{{RC}}_{{d}}}& \frac{1}{{{RC}}_{{d}}}\\ \frac{1}{{{C}}_{{f}}}& \frac{1}{{{RC}}_{{f}}}& -\frac{1}{{{RC}}_{{f}}}\end{array}\right]\left[\begin{array}{c}{{i}}_{{{L}}_{{f}}}\\ {{V}}_{{{C}}_{{d}}}\\ {{V}}_{0}\end{array}\right]{+}\left[\begin{array}{c}\frac{1}{{{L}}_{{f}}}\\ {0}\\ {0}\end{array}\right]{{V}}_{{in}}$$

37 $${{T}}\left({{s}}\right) = \frac{{{V}}_{{o}}}{{{V}}_{{in}}} = \frac{{{s}}{{R}}_{{d}}{{{C}}}_{{d}}+ {1} }{{{{s}}^{3}{{{R}}}_{{d}}{{C}}}_{{d}}{{{C}}}_{{f}}{{{L}}}_{{f}}{+}{{s}}^{2}\left({{{R}}_{{d}}{{C}}}_{{f}}{{{L}}}_{{f}}{+}{{R}}_{{d}}{{{C}}}_{{d}}{{{L}}}_{{f}}\right)+ {s} {{R}}_{{d}}{{{C}}}_{{d}}+ {1} }$$

### Π DM filter

**Figure 5:**
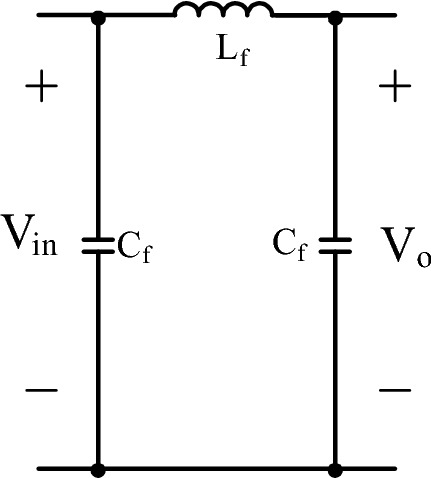
π DM filter.

pi filter is given in Fig. 5. Pi filter is similar to LC filter except it has also a filter capacitor at the input side of the filter. Filter components are calculated by using (29), (30).

Transfer function in (38) and mathematical model of the pi filter is the same as with LC filter by neglecting the input impedance.

38 $${{T}}\left({{s}}\right) = \frac{{{V}}_{{o}}}{{{V}}_{{in}}} = \frac{1}{{{s}}^{2}{{{L}}}_{{f}}{{{C}}}_{{f}}+ {1} }$$

### LCL DM filter

**Figure 6:**
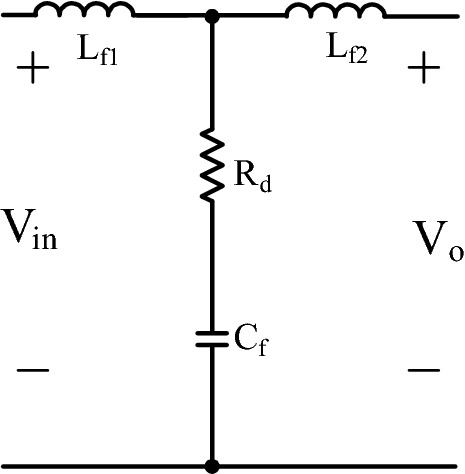
DM LCL filter.

In Fig. 6, LCL filter structure is given. LCL filter is very common in inverter and three phase rectifiers as in^8–11^. Nevertheless, LCL filter integration for PFC single phase converter is not examined in detail in literature. As an advantage, lower total inductor is required regarding to LC filter when the LCL filter is designed appropriately.

Using (39), (40), (41), (42) the filter is designed as in^8,9^. C_f_ is calculated by using (29). Frequency criteria of resonance (f_res_), grid (f_g_), and switching frequency (f_sw_) is given in (42).

39 $${{R}}_{{d}} = \frac{1}{{3\omega}_{{res}}{{{C}}}_{{f}}}$$

40 $${\omega}_{{res}} = \sqrt{\frac{{{L}}_{{f1}}{+}{{L}}_{{f2}}}{{{L}}_{{f1}}{{{L}}}_{{f2}}{{{C}}}_{{f}}}}$$

41 $${10}{{f}}_{{g}}{<}{{f}}_{{res}}{<0.5}{{f}}_{{sw}}$$

42 $${{L}}_{{f2}}= {r} {{L}}_{{f1}}$$

LCL filter’s mathematical model is obtained in (43) with i_Lf2_/V_in_ transfer function by using two port networks that is so convenient for inverter application purpose as in^8^. In order to make a comparison with other filter topologies, (44) derives filter’s V_o_/V_in_ transfer function assuming L_f2_ is added to the converter input inductance.

43 $$\dot{\left[\begin{array}{c}{{i}}_{{{L}}_{{f1}}}\\ {{i}}_{{{L}}_{{f2}}}\\ {{V}}_{{C}}\end{array}\right]}{=}\left[\begin{array}{ccc}-\frac{{{R}}_{{d}}}{{{L}}_{{f1}}}& \frac{{{R}}_{{d}}}{{{L}}_{{f1}}}& -\frac{1}{{{L}}_{{f1}}}\\ \frac{{{R}}_{{d}}}{{{L}}_{{f2}}}& -\frac{{{R}}_{{d}}}{{{L}}_{{f2}}}& \frac{1}{{{L}}_{{f2}}}\\ \frac{1}{{{C}}_{{f}}}& -\frac{1}{{{C}}_{{f}}}& {0}\end{array}\right]\left[\begin{array}{c}{{i}}_{{{L}}_{{f1}}}\\ {{i}}_{{{L}}_{{f2}}}\\ {{V}}_{{C}}\end{array}\right]{+}\left[\begin{array}{cc}\frac{1}{{{L}}_{{f1}}}& {0}\\ {0}& -\frac{1}{{{L}}_{{f2}}}\\ {0}& {0}\end{array}\right]\left[\begin{array}{c}{{v}}_{{i}}\\ {{v}}_{0}\end{array}\right]$$

44 $${{T}}\left({{s}}\right) = \frac{{{V}}_{{o}}}{{{V}}_{{in}}} = \frac{{{R}}_{{d}}{{{C}}}_{{f}}{s+1}}{{{s}}^{2}{{{C}}}_{{f}}{{{L}}}_{{f1}}+ {s} {{C}}_{{f}}{{{R}}}_{{d}}+ {1} }$$

## Applications

**Figure 7:**
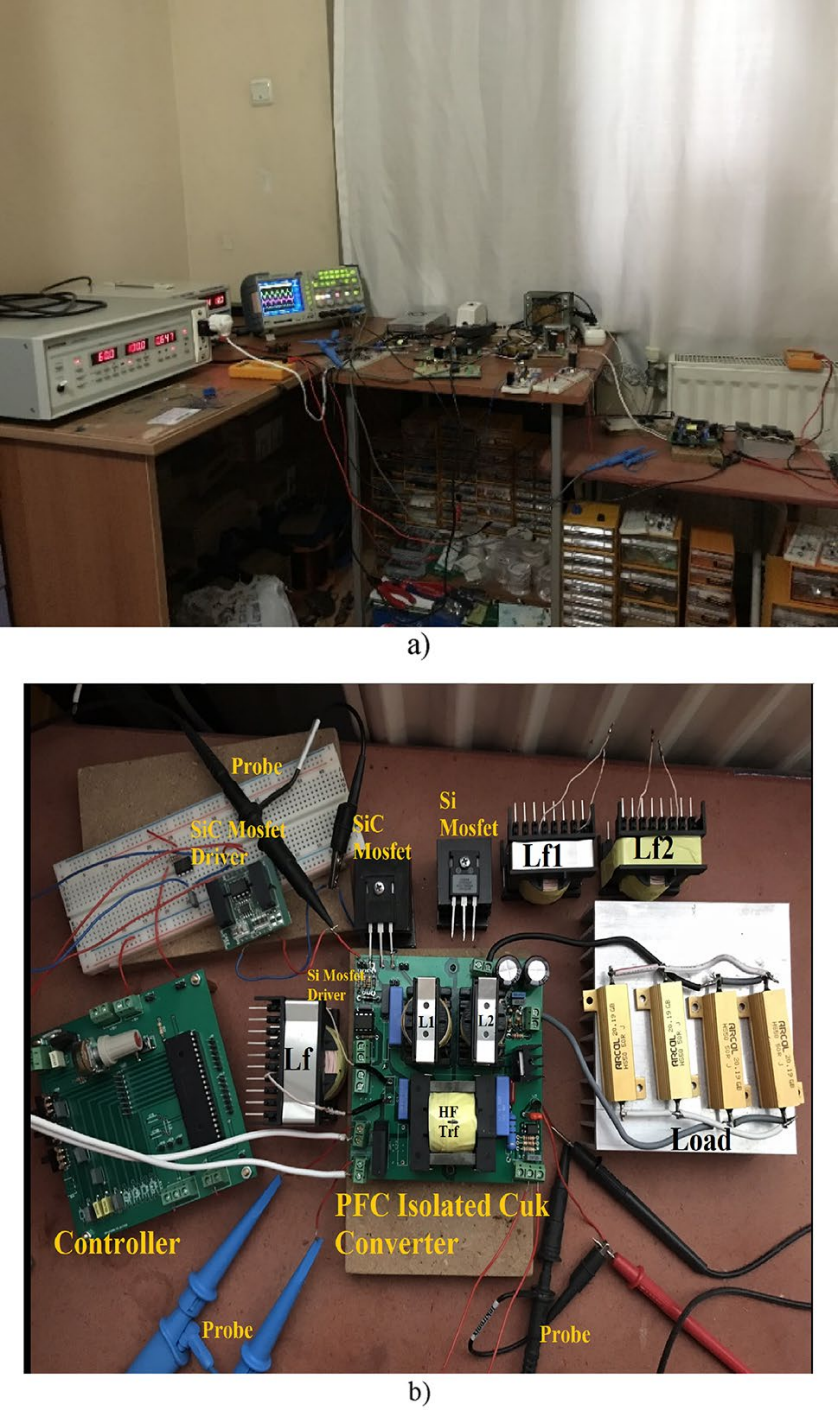
(**a**) Laboratory environment, (**b**) application circuit.

Applications are conducted with 42 kHz switching frequency and 50 W power employing each DM filters separately through isolated Ćuk PFC. Besides, as a power switch of the converter IXYS IXFH12N120P Si Mosfet and Cree C2M0280120D SiC Mosfet are employed. Moreover, each DM filter are applied and compared for both power switches. Current and voltage measurements are realized with A622, TPP0201 and P5122 probes. As a power supply APS-9501 GWInstek is used for experiments. In addition, in this study grid is considered as an ideal. Laboratory set up is also shown in Fig. 7. After calculation with (1), (2), (3), (4), (5), component values of PF isolated Ćuk converter are used these are L_1_ = 1180 μH, L_2_ = 654 μH, C_0_ = 940 μF, C_1_ = C_2_ = 1 μF. Supply of the converter is 100 V, 60 Hz grid. Besides, high frequency transformer’s turn ratio is chosen as 5 and the load at nominal operation point is 12.5 Ω.

Table 1 lists each DM filter parameters. Values are calculated and chosen with respect to (29), (30), (33), (34), (35), (39), (40), (41), (42). Although filter inductance values seem to be higher due to the low load power, by increasing filter capacitor value, lower inductance values can be used. However, extra care should be made avoiding making PF and THD values worse while increasing filter capacitor value.

**Table 1:** Passive values of DM filters.

Filter	L_f_ (L_f1_)	L_f2_	C_f_	R_d_	C_d_
LC	35 mH	–	22 nF	–	–
π	35 mH	–	22 nF	–	–
LC with damping	35 mH	–	22 nF	7.7 kΩ	88 nF
LCL	9 mH	10.2 mH	22 nF	155 Ω	-

Before analyzing the effect of DM filter structures, isolated PFC Ćuk converter's transfer function is derived in (45) with the values used in the application through (28). Each filter’s transfer function can be cascaded to (45), and this make the analysis with linear methods possible.

45 $${{T}}\left({{s}}\right) = \frac{\widetilde{{{V}}_{0}}}{{d}} = \frac{{8.77}  \times  {10}^{7}{{{s}}}^{3}{+6.042 \times }{10}^{12}{{{s}}}^{2}{+4.302 \times }{10}^{17}{{s}}}{{{s}}^{5}{+85.11}{{s}}^{4}{+4.464 \times }{10}^{9}{{{s}}}^{3}{+3.797 \times }{10}^{11}{{{s}}}^{2}{+7.607 \times }{10}^{15}{{s}}}$$

**Figure 8:**
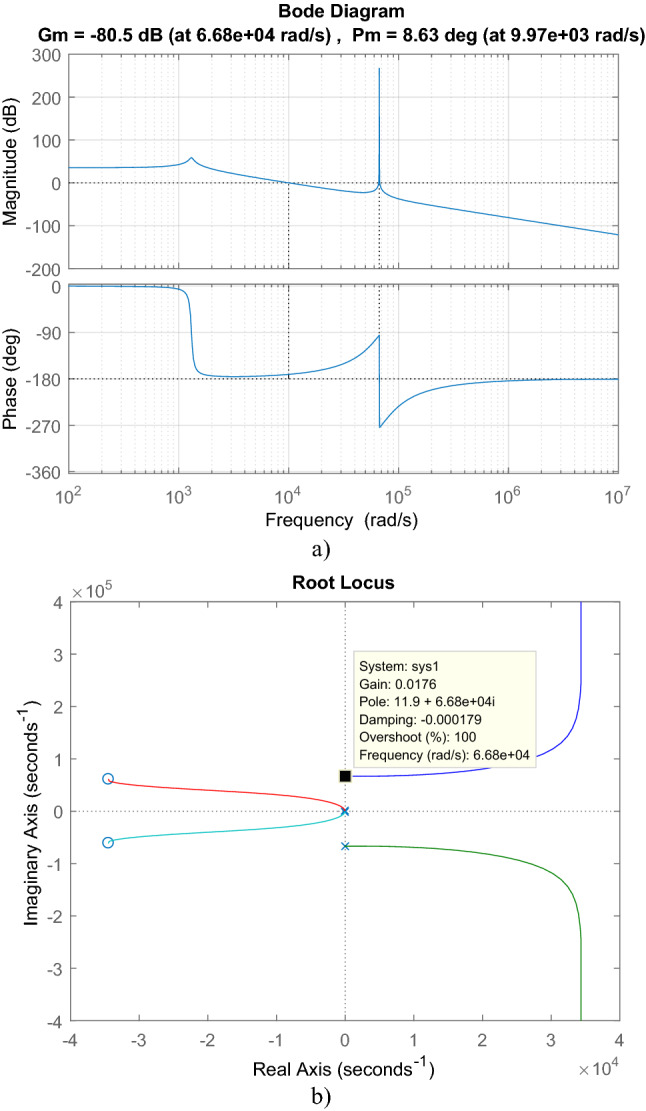
For isolated PFC Ćuk converter (**a**) Bode, (**b**) root locus graphs.

With the transfer function in (45), bode and root locus plot of isolated PFC Ćuk converter are sketched in Fig. 8. 8.63° phase margin and -80.5 dB gain margin are obtained by bode graph. From root locus plot, the converter has four poles located at − 1.4211 × 10^4^ ± 6.6797 × 10^4^j, − 42.553 ± 1.3050 × 10^3^j and two zeros located at − 3.4462 × 10^4^ ± 6.0995 × 10^4^j. Gain should be lower than 9.387 × 10^–5^ to provide the stability.

### LC DM filter

LC filter is connected to the isolated PFC Ćuk converter’s input, so they have a cascade connection. By considering transfer functions in (32) and (45), overall transfer function is derived in (46). Sketching root locus and bode plots which are linear methods, impact of the filter to the converter is analyzed with respect to control point of view.

46 $${{T}}\left({{s}}\right) = \frac{\widetilde{{{V}}_{0}}}{{d}} = \frac{{8.77 \times }{10}^{7}{{{s}}}^{3}{+6.042 \times }{10}^{12}{{{s}}}^{2}{+4.302 \times }{10}^{17}{{s}}}{\begin{array}{c}{7.7}{{ \times }}{10}^{-10}{{s}}^{7}{+}{6.55}{{ \times }}{10}^{-8}{{s}}^{6}{+}{4.437}{{s}}^{5}{+}{377.5}{{s}}^{4}{+}\\ {4.47}{{ \times }}{10}^{9}{{{s}}}^{3}{+}{3.797}{{ \times }}{10}^{11}{{{s}}}^{2}{+}{7.607}{{ \times }}{10}^{15}{{s}}\end{array}}$$

**Figure 9:**
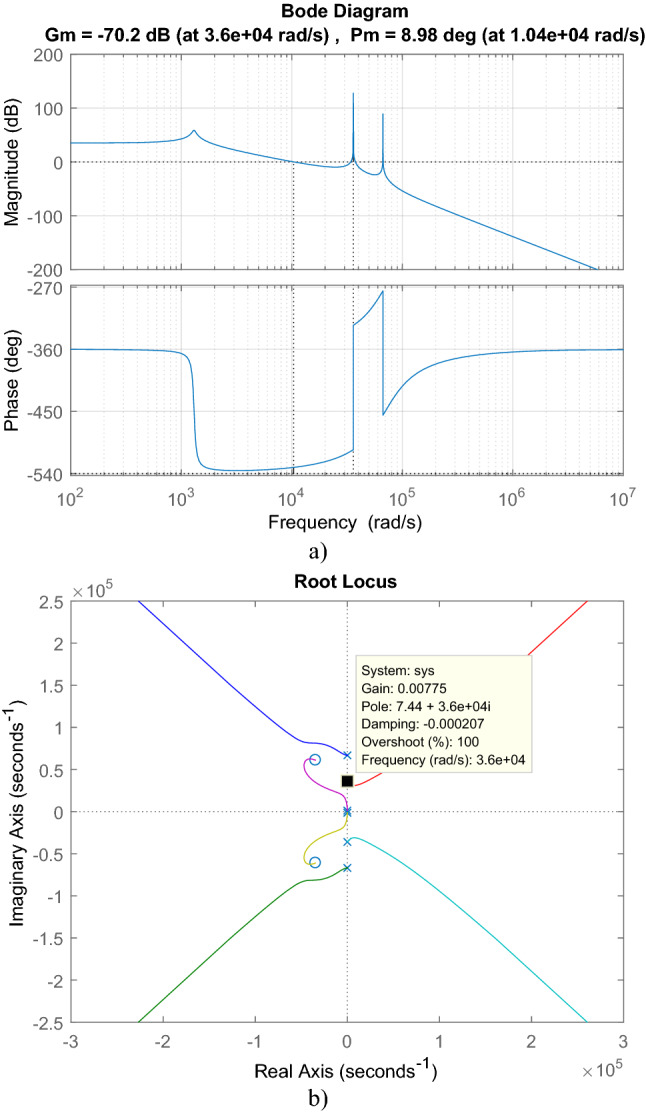
For LC DM filter with isolated Ćuk PFC (**a**) Bode diagram, (**b**) root locus graph.

For cascaded transfer function in (46), bode plot in Fig. 9a, root locus in Fig. 9b are depicted. From bode plot, the cascaded transfer function has 8.98° phase margin and − 70.2 dB gain margin. By root locus, LC filter with converter has two zeros located at − 3.4463 × 10^4^ ± 6.0991 × 10^4^j and six poles located at − 9.6344 × 10^–3^ ± 6.68 × 10^4^j, − 1.14 × 10^–13^ ± 3.6 × 10^4^j, − 42.545 ± 1.3050 × 10^3^j. Also, 3.0894 × 10^–4^ is the gain limit for instability.

**Figure 10:**
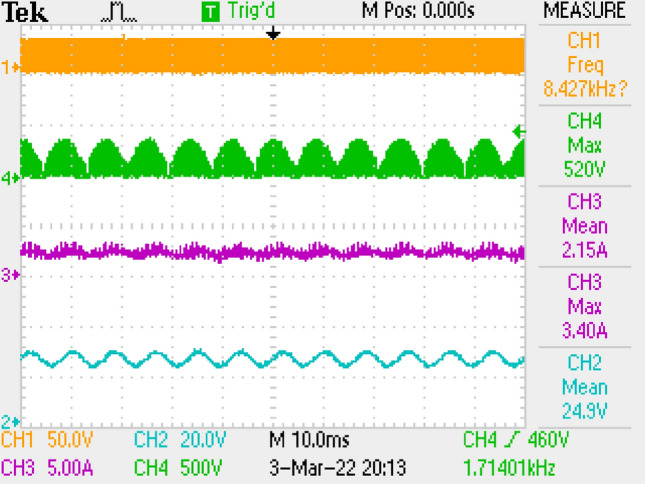
Measurements by LC DM filter, PWM, voltage of switch voltage, current and voltage of output.

**Figure 11:**
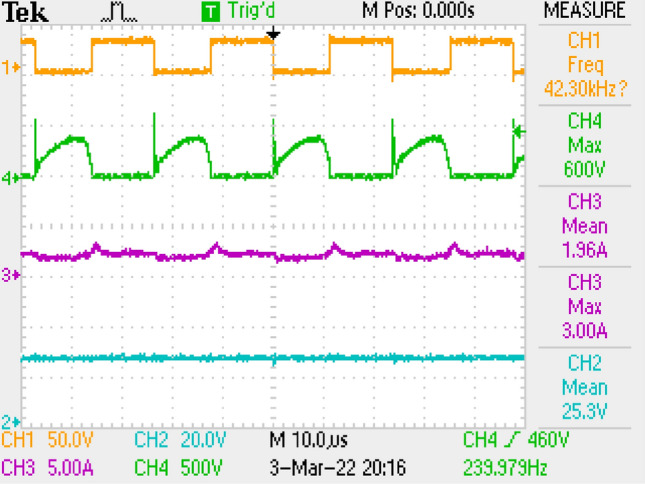
By LC DM filter, (zoomed of Fig. 10).

Measurement results by employing LC filter is shown in Fig. 10 covering PWM signal, output current, switch voltage, output voltage. Figure 11 is the zoomed figure of Fig. 10. It is seen by the figures that because of the leakage inductance of the converter, switch voltage has a 600 V peak. Also, output voltage is around 24.9 V, output current is around 2.15 A with 120 Hz ripple frequency which is twice of the grid frequency. Besides, duty cycle (D) is around 46.9% with 42 kHz switching frequency.

**Figure 12:**
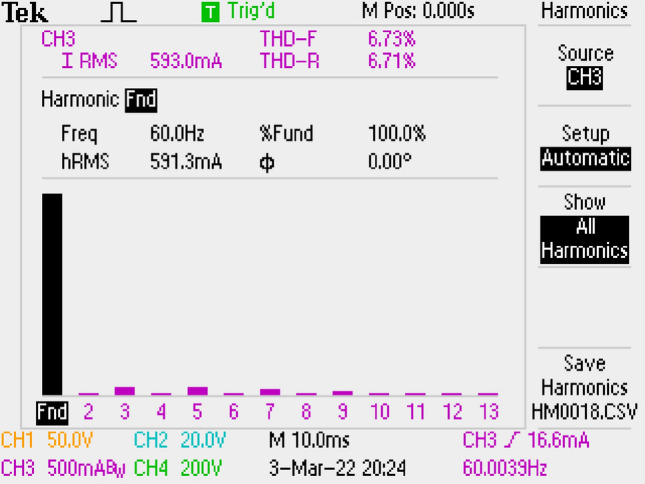
By LC DM filter, THD of grid current.

Grid current THD’s is conducted as 6.73% and shown in Fig. 12.

**Figure 13:**
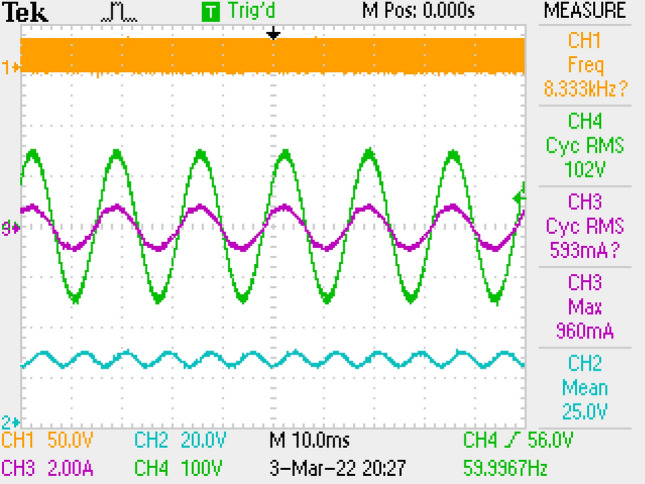
PWM, voltage-current of grid, output voltage, by LC DM filter.

PWM, voltage of output, current–voltage of grid are given in Fig. 13. PF is also measured as 0.997.

### LC DM filter with parallel damping

Grid connection of isolated PFC Ćuk converter is provided by LC with parallel damping filter so they have a cascade connection. By considering transfer functions in (37) and (45), overall transfer function in (47) is derived. Sketching root locus and bode plots of (47) which are linear methods, impact of the filter to the converter is analyzed with respect to control point of view.

47 $${{T}}\left({{s}}\right) = \frac{\widetilde{{{V}}_{0}}}{{d}} = \frac{{5.94 \times }{10}^{4}{{{s}}}^{4}{+4.182 \times }{10}^{9}{{{s}}}^{3}{+2.975 \times }{10}^{14}{{{s}}}^{2}{+4.302 \times }{10}^{17}{{s}}}{\begin{array}{c}{5.22}{{ \times }}{10}^{-13}{{s}}^{8}{+}{2.96}{{ \times }}{10}^{-5}{{s}}^{7}{+}{0.00553}{{s}}^{6}{+}{1.32}{{{ \times 10}}^{5}{{s}}}^{5}{+}\\ {1.4}{{ \times }}{10}^{7}{{{s}}}^{4}{+}{2.3}{{ \times }}{10}^{11}{{{s}}}^{3}{+}{5.5}{{ \times }}{10}^{12}{{{s}}}^{2}{+}{7.6}{{ \times }}{10}^{15}{{s}}\end{array}}$$

**Figure 14:**
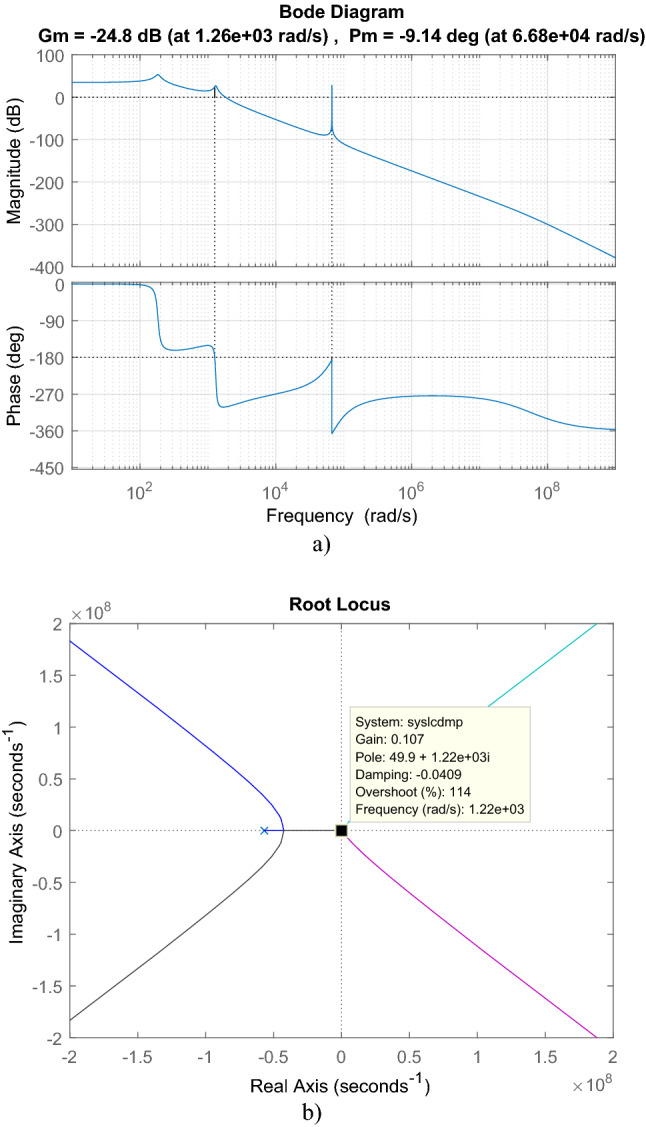
For LC with damping DM filter with isolated Ćuk PFC, (**a**) Bode diagram, (**b**) root locus.

For cascaded transfer function in (47), bode plot in Fig. 14a, root locus in Fig. 14b are depicted. From root locus, it has three zeros located at − 3.4463 × 10^4^ ± 6.099 × 10^4^j, − 1.4758 × 10^3^ and seven poles located at − 5.6818 × 10^7^, − 9.6344 × 10^–3^ ± 6.68 × 10^4^j, − 42.545 ± 1.3050 × 10^3^j, − 14.28 ± 183,31j. Maximum gain for the stability is 0.0576. From bode plot, the cascaded transfer function has − 9.14° phase margin and − 24.8 dB gain margin.

**Figure 15:**
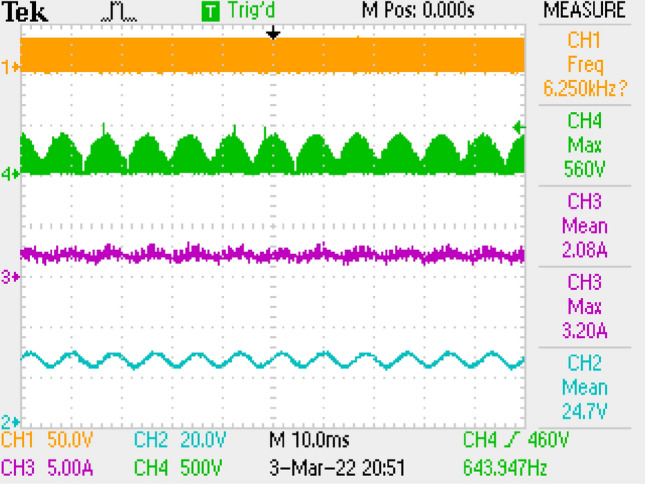
Measurements by LC with parallel damping DM filter, PWM, voltage of switch, current–voltage of output.

**Figure 16:**
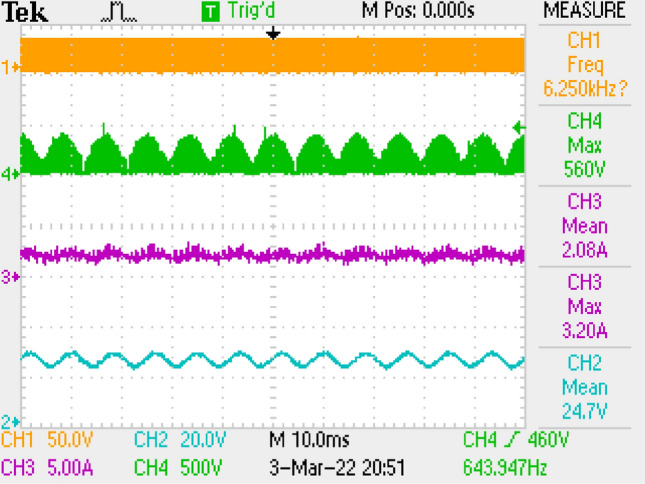
By LC with parallel damping DM filter, (zoomed of Fig. 15).

Figure 15 gives PWM, current of output, voltages of switch and output. Figure 16 is the zoomed figure of Fig. 15. It is observed by the figures that because of the leakage inductance of the converter, switch voltage has a 600 V peak. Also, output voltage is around 24.7 V, output current is around 2.08 A with 120 Hz ripple frequency which is twice of grid frequency. Besides, duty cycle (D) is around 47% with 42 kHz switching frequency.

**Figure 17:**
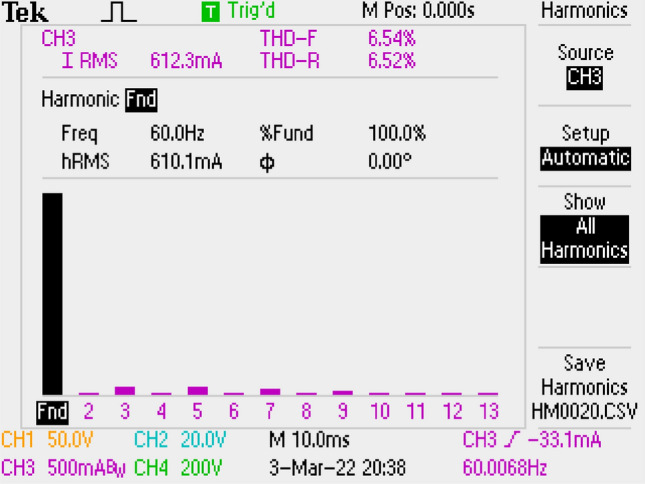
By LC with damping DM filter, THD of grid current.

Grid current THD’s is conducted as 6.54% and shown in Fig. 17.

**Figure 18:**
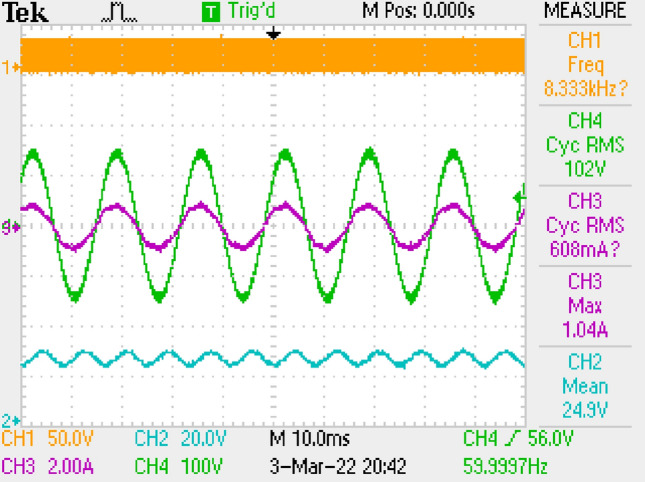
PWM, voltage-current of grid, output voltage by LC with parallel damping DM filter.

PWM signal, output voltage, current–voltage of grid are depicted in Fig. 18. PF is also measured as 0.997.

### Π DM filter

Cascaded transfer function is the same as LC filter with isolated Ćuk PFC given in (46). As LC filter, pi filter has also the same characteristic.

**Figure 19:**
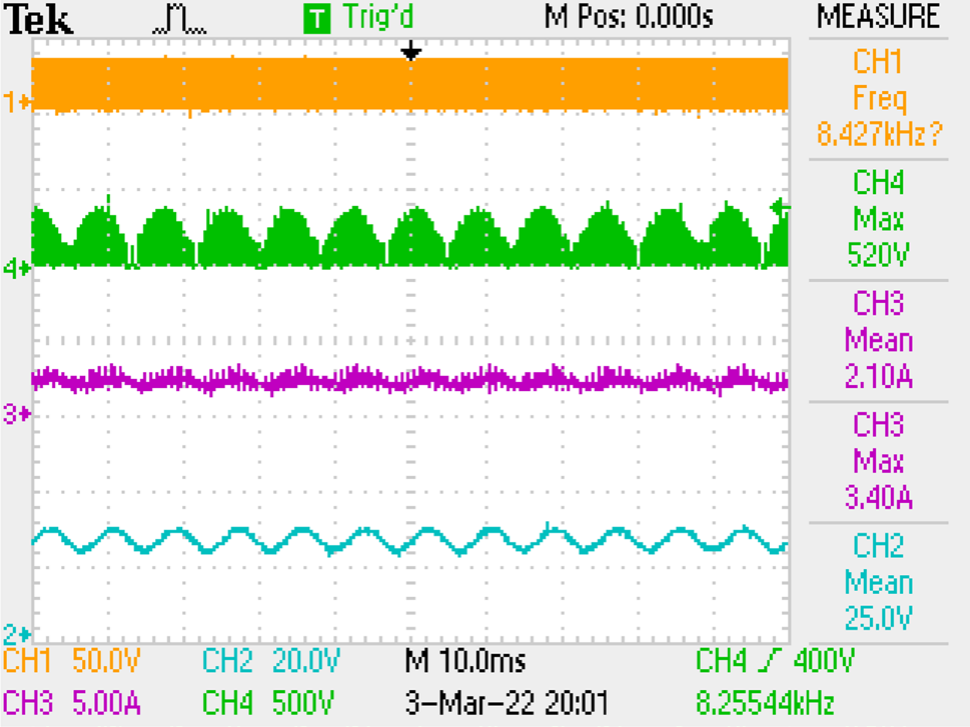
By π DM filter, PWM, voltage of switch, current–voltage of output.

**Figure 20:**
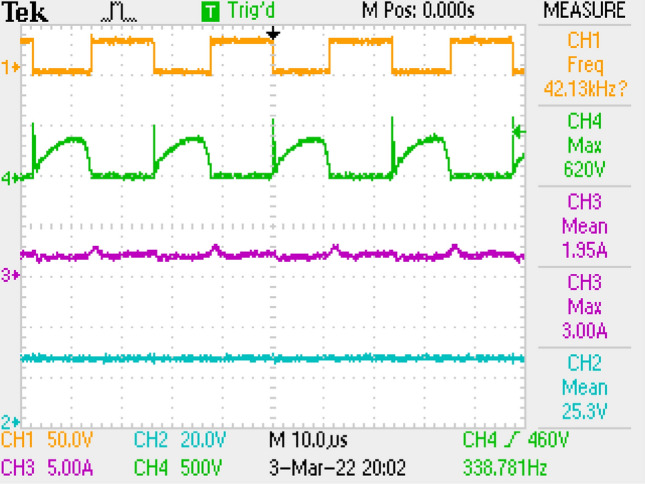
By π DM filter, (zoomed of Fig. 19).

PWM, output current, voltage of switch, output voltage is shown for pi filter in Fig. 19. Figure 20 is the zoomed figure of Fig. 19. It is concluded by the figures that because of the leakage inductance of the converter, switch voltage has a 620 V peak. Also, output current is around 2.1 A with 120 Hz ripple frequency which is twice of grid frequency, output voltage is around 25 V. Besides, duty cycle (D) is around 46.9% with 42 kHz switching frequency.

**Figure 21:**
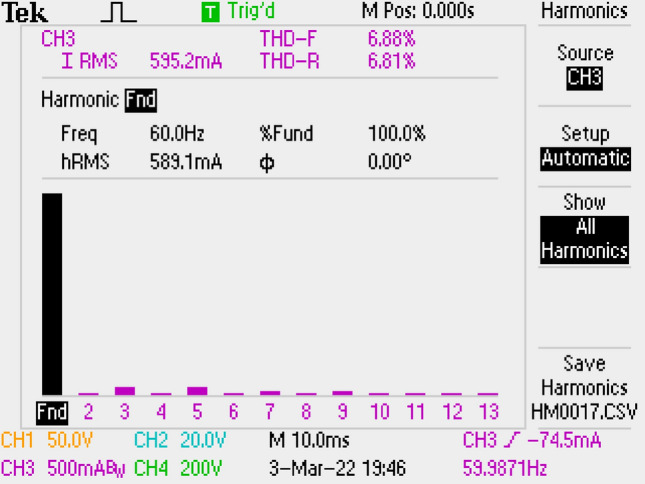
By π DM filter, grid current THD.

Grid current has 6.88% THD and shown in Fig. 21.

**Figure 22:**
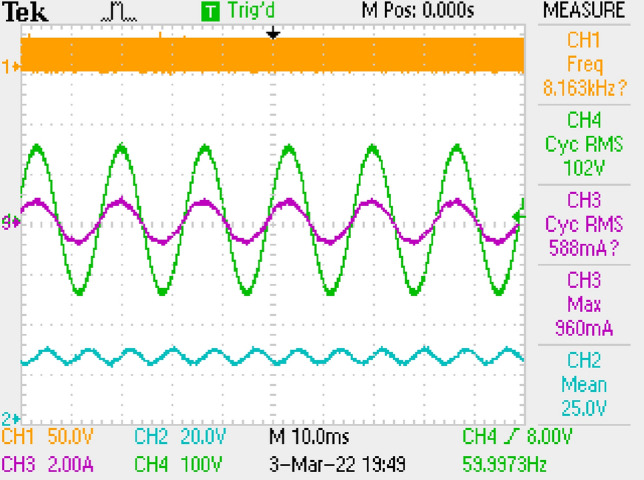
PWM, voltage-current of grid, voltage of output by π DM filter.

Figure 22 gives PWM, voltage of output, voltage-current of grid. PF is measured as 0.998.

### LCL DM filter

**Figure 23:**
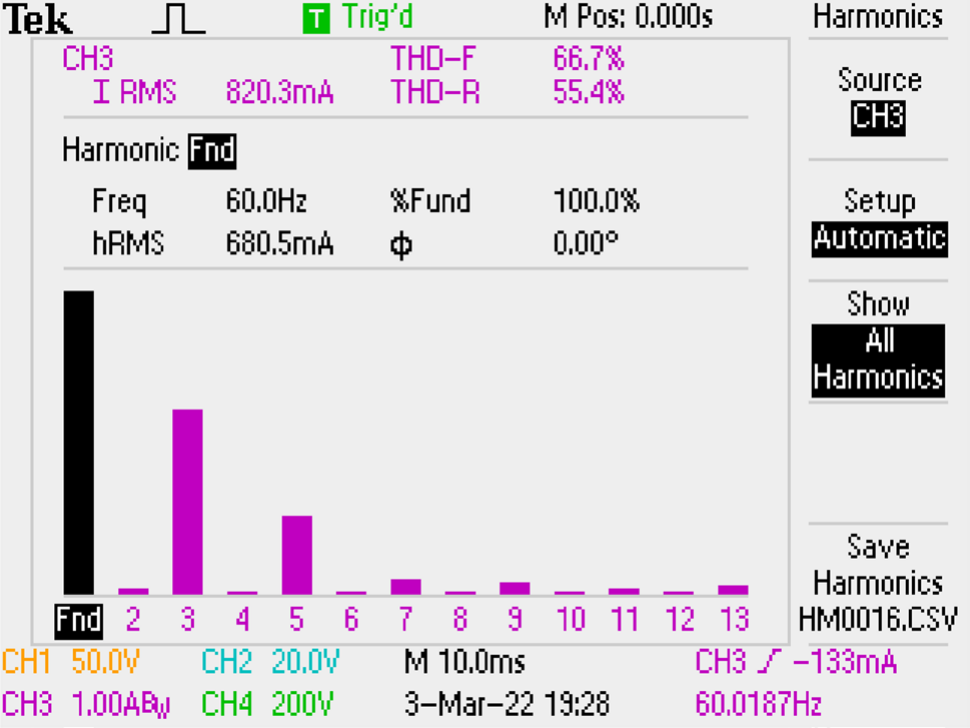
By LCL DM filter, THD of grid current.

**Figure 24:**
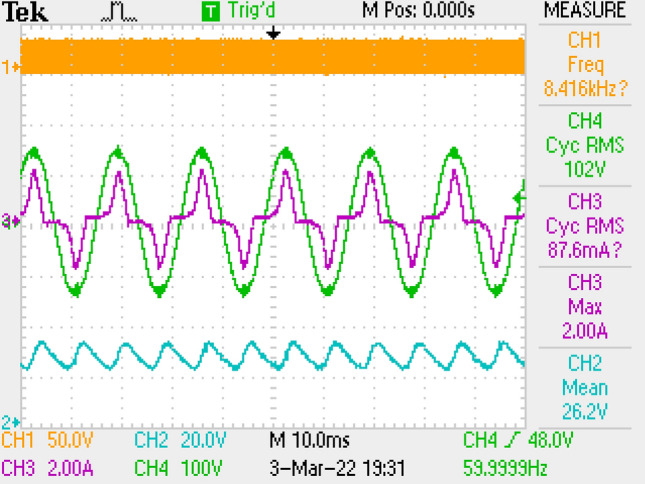
PWM, grid voltage-current, voltage of output, by LCL DM filter.

Results of application are given in Figs. 23, 24 by using LCL filter solely. PF as 0.821 and THD as 66.7% are measured. These values are not acceptable for the standards. The result is because of the filter inductor L_f2_ that increases the converter’s total input inductor value. The total input inductor is then found as 11.28 mH which is higher than (1). That causes in the change of the operation of the converter to CCM. For CCM mode of operation, to increase PF and THD, another control algorithms should be used which is more complex.

**Figure 25:**
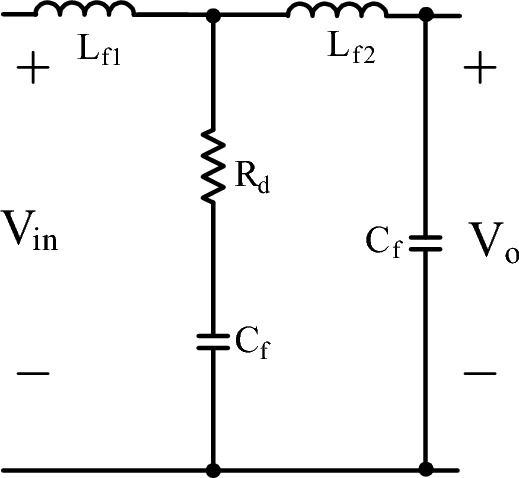
LCL with parallel C DM filter.

To increase the effectivity of LCL filter, another capacitor with the same capacitance of C_f_ is placed to the input of the converter. Although LCL filter is used for single phase PWM rectifier with additional LC structure in^37^ which is the only study that uses LCL filter for PFC rectifier, the analysis of the filter is not given in detail. So, the new proposed LCL filter type with parallel capacitor is sketched in Fig. 25. Filter values are the same as of LCL DM filter. Also, (48) derives filter transfer function.

48 $${{T}}\left({{s}}\right) = \frac{{{V}}_{{o}}}{{{V}}_{{in}}} = \frac{{{R}}_{{d}}{{{C}}}_{{f}}{{{L}}}_{2}{s} + {{L}}_{2}}{\begin{array}{c}{{s}}^{4}{{{C}}_{{f}}}^{2}{{{L}}_{{f2}}}^{2}{{{L}}}_{{f1}}{+}{{s}}^{3}\left({{{C}}_{{f}}}^{2}{{{L}}_{{f2}}}^{2}{{{R}}}_{{d}}{+}{{{C}}_{{f}}}^{2}{{{L}}}_{{f2}}{{{L}}}_{{f1}}{{{R}}}_{{d}}\right){+}\\ {{s}}^{2}\left({{{L}}_{{f2}}}^{2}{{{C}}}_{{f}}{+}{{L}}_{{f2}}{{{L}}}_{{f1}}{{{C}}}_{{f}}\right){+}{{s}}{{L}}_{{f2}}{{{R}}}_{{d}}{{{C}}}_{{d}}{+}{{L}}_{{f2}}\end{array}}$$

LCL filter with parallel C has also cascade connection with PFC isolated Ćuk converter, so the transfer function of cascade connection is obtained as in (49) for examining the impact of the filter. Also, (50) presents the mathematical model of the LCL with C filter.

49 $${{T}}\left({{s}}\right) = \frac{\widetilde{{{V}}_{0}}}{{d}} = \frac{{2.99}{{s}}^{4}{+1.083 \times }{10}^{6}{{{s}}}^{3}{+7.509 \times }{10}^{10}{{{s}}}^{2}{+4.302 \times }{10}^{15}{{s}}}{\begin{array}{c}{4.36}{{ \times }}{10}^{-22}{{s}}^{9}{+}{1.43}{{ \times }}{10}^{-17}{{s}}^{8}{+}{8.1}{{ \times }}{10}^{-12}{{s}}^{7}{+}{9.842}{{ \times }}{10}^{-8}{{s}}^{6}{+}\\ {0.0365}{{s}}^{5}{+}{155.4}{{s}}^{4}{+}{4.024}{{ \times }}{10}^{7}{{{s}}}^{3}{+}{3.68}{{ \times }}{10}^{9}{{{s}}}^{2}{+}{6.85}{{ \times }}{10}^{13}{{s}}\end{array}}$$

50 $$\dot{\left[\begin{array}{c}{{i}}_{{{L}}_{{f1}}}\\ {{i}}_{{{L}}_{{f2}}}\\ {{V}}_{{C}}\\ {{V}}_{{o}}\end{array}\right]} = \left[\begin{array}{cccc}\frac{-{{R}}_{{d}}}{{{L}}_{{f1}}}& \frac{{{R}}_{{d}}}{{{L}}_{{f1}}}& \frac{-1}{{{L}}_{{f1}}}& {0}\\ \frac{{{R}}_{{d}}}{{{L}}_{{f2}}}& \frac{{{-R}}_{{d}}}{{{L}}_{{f2}}}& \frac{1}{{{L}}_{{f2}}}& \frac{-1}{{{L}}_{{f2}}}\\ \frac{1}{{{C}}_{{f}}}& \frac{-1}{{{C}}_{{f}}}& {0}& {0}\\ {0}& \frac{1}{{{C}}_{{f}}}& {0}& {0}\end{array}\right]\left[\begin{array}{c}{{i}}_{{{L}}_{{f1}}}\\ {{i}}_{{{L}}_{{f2}}}\\ {{V}}_{{C}}\\ {{V}}_{0}\end{array}\right]{+}\left[\begin{array}{c}\frac{1}{{{L}}_{{f1}}}\\ {0}\\ {0}\\ {0}\end{array}\right]{{v}}_{{i}}$$

**Figure 26:**
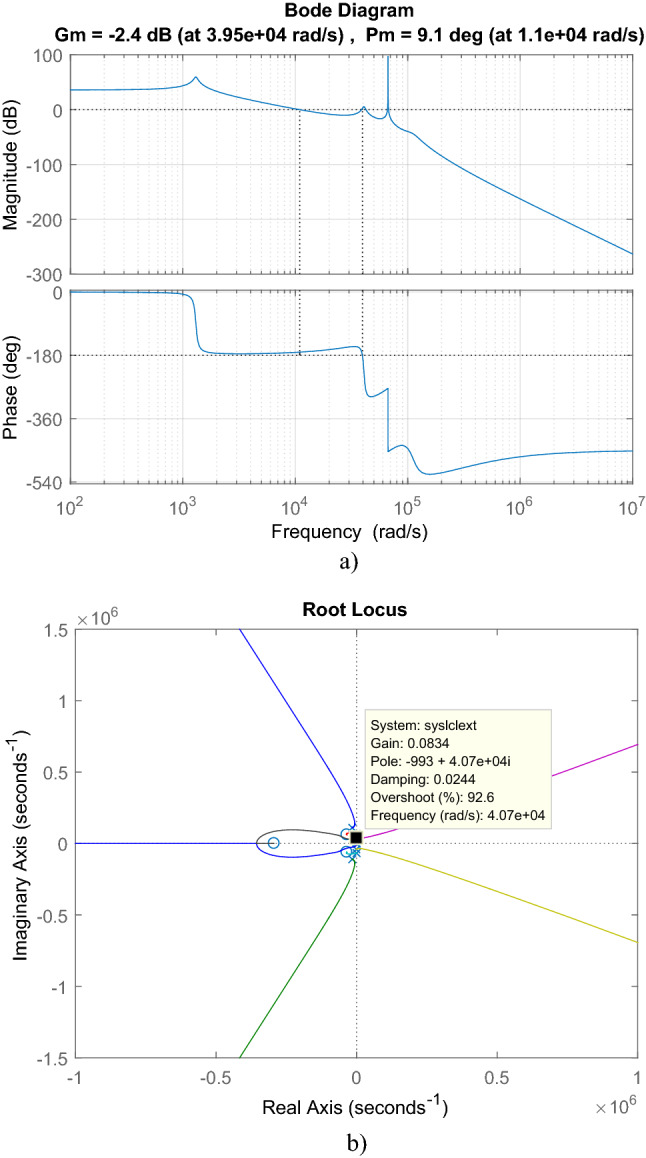
For LCL with parallel C DM filter with isolated Ćuk PFC (**a**) Bode diagram, (**b**) root locus.

For cascaded transfer function in (49), bode plot in Fig. 26a, root locus in Fig. 26b are depicted. By root locus plot the cascaded transfer function has three zero located at − 3.4463 × 10^4^ ± 6.099 × 10^4^j, − 2.9326 × 10^5^ and eight poles located at − 9.6344 × 10^–3^ ± 6.68 × 10^4^j, − 42.545 ± 1.3050 × 10^3^j, − 1.5254 × 10^4^ ± 1.1035 × 10^5^j, − 1.1076 × 10^3^ ± 4.079 × 10^4^j. The gain limit for instability is obtained as 0.7585. From bode plot, the cascaded transfer function has 9.1° phase margin and -2.4 dB gain margin.

**Figure 27:**
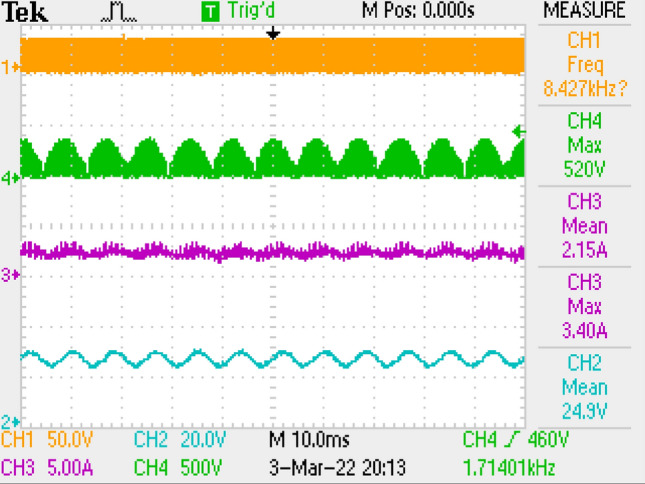
By LCL with C DM filter, PWM, voltage of switch, current–voltage of output.

**Figure 28:**
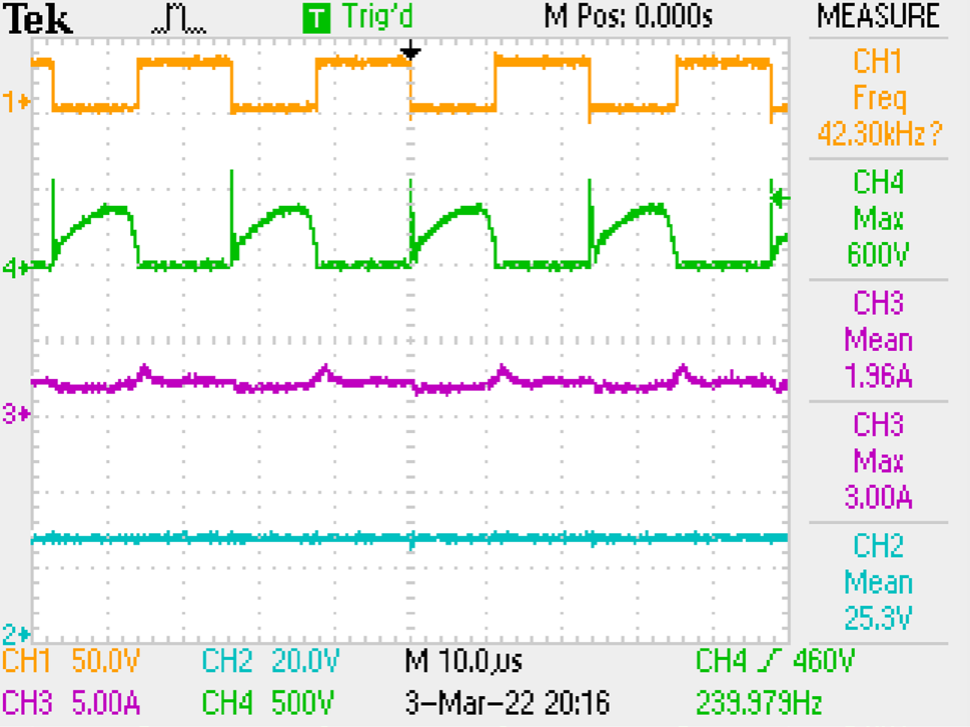
By LCL with C DM filter, (zoomed of Fig. 27).

Also, application results are given with respect to PWM signal, output current, switch voltage, output voltage in Fig. 27. Figure 28 is the zoomed figure of Fig. 27. It is observed by the figures that because of the leakage inductance of the converter, switch voltage has a 620 V peak. Also, output voltage is around 24.9 V, output current is around 2.15 A with 120 Hz ripple frequency which is twice of grid frequency. Besides, duty cycle (D) is around 36.5% with 42 kHz switching frequency.

**Figure 29:**
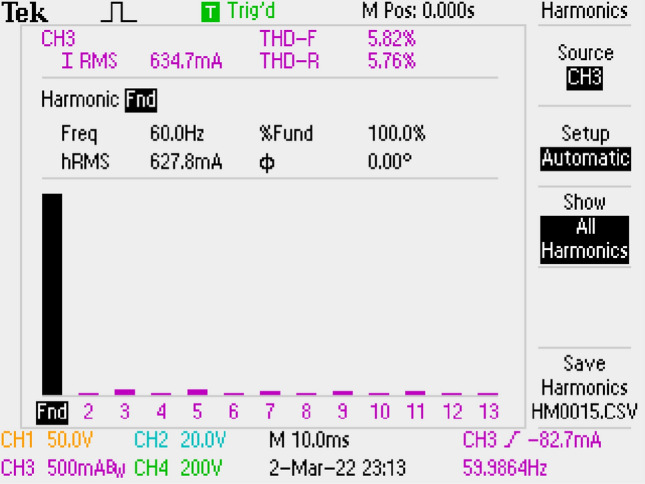
By LCL with C DM filter, THD of grid current with.

Grid current THD’s is given in Fig. 29. THD is measured as 5.82%.

**Figure 30:**
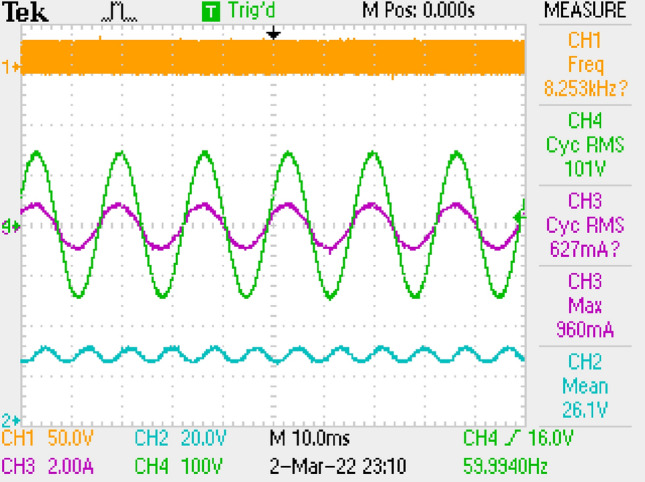
PWM, voltage-current of grid, voltage of output, by LCL with C DM filter.

Figure 30 gives PWM, voltage of output and voltage-current of grid. PF is also measured as 1.

**Figure 31:**
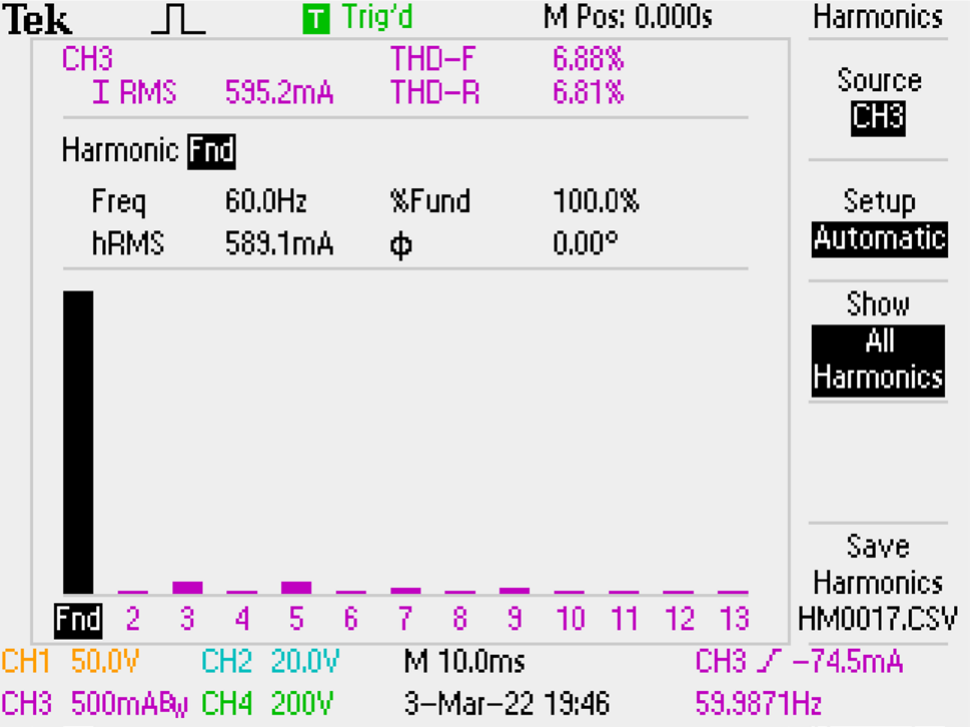
By LCL with C DM filter, THD of grid current, for Si Mosfet.

THD of grid current is measured as 4.90% by using Si Mosfet. It is shown in Fig. 31 which is better than the result of SiC Mosfet.

**Figure 32:**
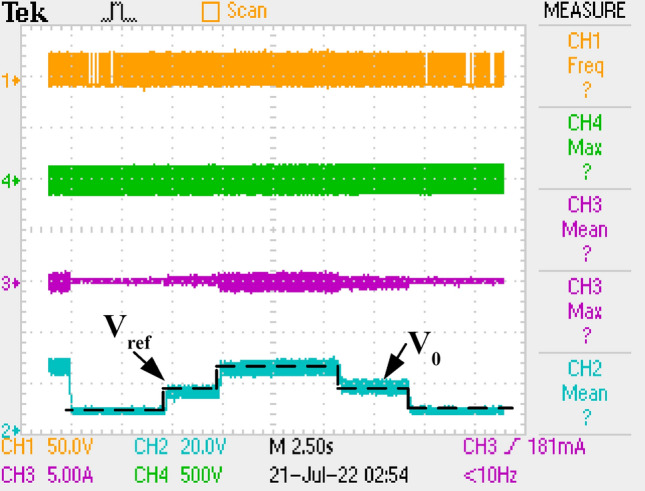
Output Voltage of LCL with C DM filter under reference changes.

In Fig. 32, application result of output voltage regulation by using PI controller for LCL with parallel C DM filter is given under reference voltage change. Feedback signal of the converter is taken by TC431 and PC837 IC as seen in Fig. 7b. Reference voltage is changed to 5 V, 15 V, 25 V, 15 V, 5 V and the reference voltage is provided by output voltage. Besides, similar voltage control characteristics is obtained with other DM filter topologies by PI controller.

## Loss calculations

**Figure 33:**
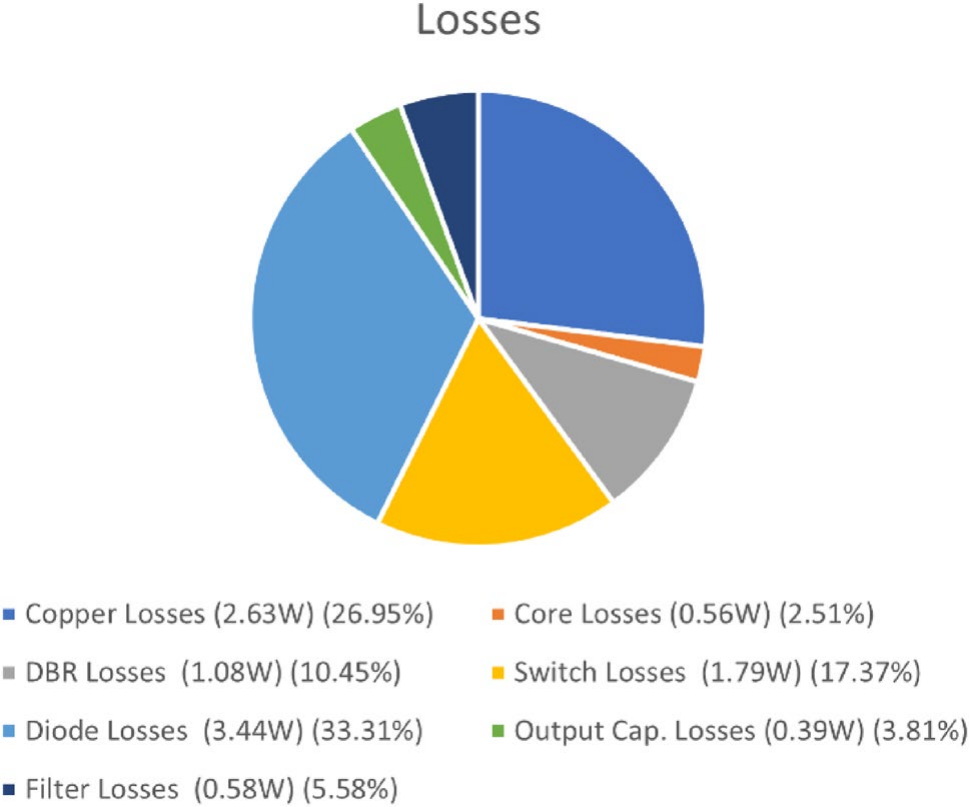
Power Losses of isolated PFC Ćuk converter.

Figure 33 presents power loss graph of the study with filter. 10.162 W is the total losses. By using the losses, the efficiency can be calculated as 83%.

Calculation of the losses are realized under seven class, these are (DBR) diode bridge, magnetic core, output diode, copper, filter, switch, output capacitor as in^45^. Calculations are accomplished with (51), (52), (53), (54), (55), (56), (57), (58), (59), (60), (61), (62), (63), (64), (65), (66), (67). SiC Mosfet is used as a power switch with 300 × 10^−3^Ω R_dson_.

51 $${{P}}_{{L1}} = {{I}}_{{L1rms}}^{2}  \times  {{DCR}}_{{L1}} = {0.6}^{2}{ \times 0.5=0.18W}$$

52 $${{P}}_{{L2}} = {{I}}_{{L2rms}}^{2}{{ \times }}{{DCR}}_{{L2}} = {2}^{2}{ \times 0.2=0.8W}$$

53 $${{P}}_{{prtrf}} = {{I}}_{{prrms}}^{2}{{ \times }}{{DCR}}_{{prm}} = {1.68}^{2}{ \times 0.4=1.13W}$$

54 $${{P}}_{{scdrtrf}} = {{I}}_{{scdrms}}^{2}{{ \times }}{{DCR}}_{{scdr}} = {8.3}^{2}{ \times 0.01=0.68W}$$

55 $${{P}}_{{corepk}} = {{\Delta B}}^{2}{{ (}\frac{{f}}{{10}^{3}}{)}}^{1.46}{{ \times }}{{V}}_{{e}}{{ \times }}{10}^{-6}{, }{{P}}_{{core}} = {{P}}_{{corepk}}\frac{2}{{\pi}}$$

56 $${{P}}_{{coreL1pk}} = {0.2}^{2}{{(}\frac{42000}{{10}^{3}}{)}}^{1.46}{{ \times 7630 \times }}{10}^{-6}{=0.0715W}{, }{{P}}_{{coreL1}}{=0.04556W}$$

57 $${{P}}_{{coreL2pk}} = {0.2}^{2}{\left (\frac{42000}{{10}^{3}}\right)}^{1.46}{{ \times 5350 \times}}{10}^{-6}{=0.050W}, \;\; {{P}}_{{coreL2}}{=0.0319W}$$

58 $${{P}}_{{coretrfpk}} = {0.4}^{2}{\left(\frac{42000}{{10}^{3}}\right)}^{1.46}{{ \times 7630 \times}}{10}^{-6}{=0.286W}, \;\; {{P}}_{{coretrf}}{=0.182W }$$

59 $${{P}}_{{DBR}} = {{2 \times I}}_{{av}}{{ \times }}{{V}}_{{f,brdg}}{=2 \times 0.636 \times \sqrt 2 \times 0.6 \times 1=1.08W}$$

60 $${{P}}_{{swtch,cond}} = {{I}}_{{swrms}}^{2}{{ \times }}{{R}}_{{dson}}= { } {1.8}^{2}{{ \times 300 \times }}{10}^{-3}{=0.972W}$$

61 $${{P}}_{{swtch,snub}} = {{Vin}}^{2}{{ \times f \times }}{{C}}_{{snub}}= { } {140}^{2}{{ \times 42000 \times }}{10}^{-9}{=0.82W}$$

62 $${{P}}_{{diode,cond}} = {{I}}_{{diode}}{{ \times }}{{V}}_{{f,diode}}{= 2 \times 1.5=3W}$$

$${{P}}_{{diode,snub}} = {{V}}_{{diode}}^{2}{{ \times f \times }}{{C}}_{{snub}}$$

63 $$= { } {76}^{2}{{ \times 42000 \times }}{{1.8 \times 10}}^{-9}{=0.436W}$$

64 $${{P}}_{{cap}} = {{I}}_{{cap}}^{2}{ \times ESR} = {1.18}^{2}{ \times 0.282=0.393W}$$

65 $${{P}}_{{Lf}} = {{I}}_{{Lfrms}}^{2}{{ \times }}{{DCR}}_{{Lf}} = {0.6}^{2}{ \times 1.6=0.576W}$$

66 $${{P}}_{{Lf1}} = {{I}}_{{Lf1rms}}^{2}{{ \times }}{{DCR}}_{{Lf1}} = {0.6}^{2}{ \times 0.3=0.108W}$$

67 $${{P}}_{{Lf2}} = {{I}}_{{Lf2rms}}^{2}{{ \times }}{{DCR}}_{{Lf2}} = {0.6}^{2}{ \times 1.1=0.396W}$$

## Results

**Figure 34:**
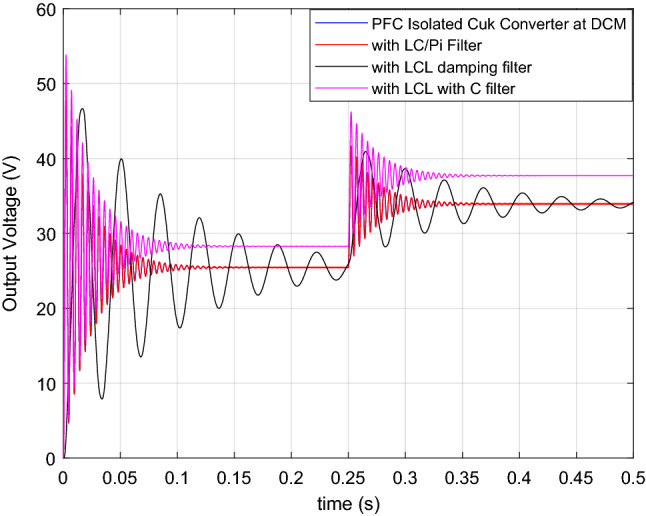
Step responses of isolated PFC Ćuk converter and with each DM filter structures.

Transfer functions are presented for isolated Ćuk PFC in (45), and LC filter in (46), LC with damping in (47), LCL with C in (49). They are approved by step responses in Fig. 34. In Fig. 34, d is adjusted as 0.45 at initial and it is changed to 0.5 at 0.25 s. Converter, with LC, with pi and with LC filter with damping gives the same output voltage, that is 25.5 V till 0.25 s after that it is 34 V. Besides, LCL with C filter has 28.3 V till 0.25 s after that it is 37.7 V. Therefore, it is proved by step responses that all models related with filters and isolated PFC Ćuk converter is accurate. Also, analysis made in this paper are validated. However, characteristics of the step response is damped oscillation, it is due to the open loop transfer functions of (45), (46), (47), (49). After closed loop compensator design for the given transfer function, such oscillation will be cancelled. Controller design is not included in the paper. In present paper, Fig. 34 is given to validate the mathematical model of the converter itself and including filters. Having similar step responses validates the accuracy of the modeling.

**Figure 35:**
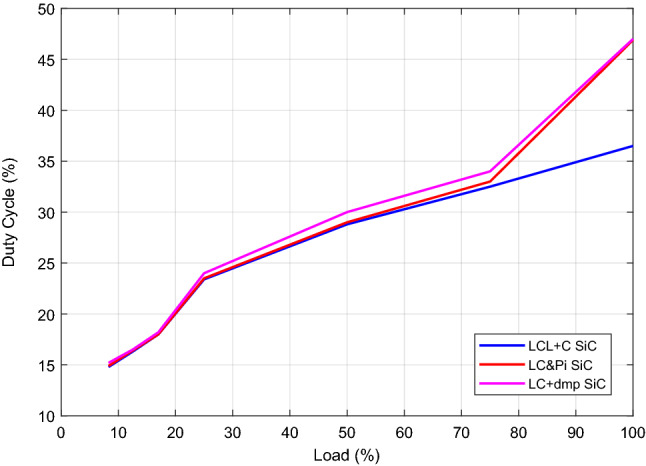
Load change versus duty cycle.

It is also observed by applications that the variation of the duty cycle proves the results in Fig. 34. The duty cycle change is given in Fig. 35 by load change with respect to the filter types. It is observed that LCL with C filter ensures the same output voltage with lower ‘d’ comparing to other filter types.

**Figure 36:**
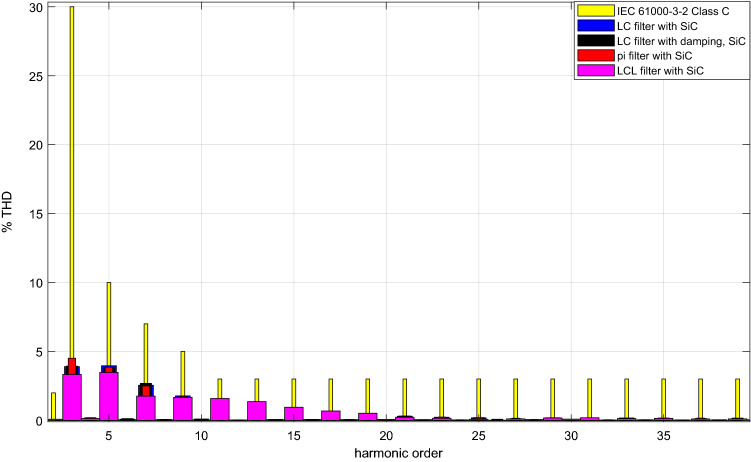
Grid current THD comparison with SiC Mosfet.

**Figure 37:**
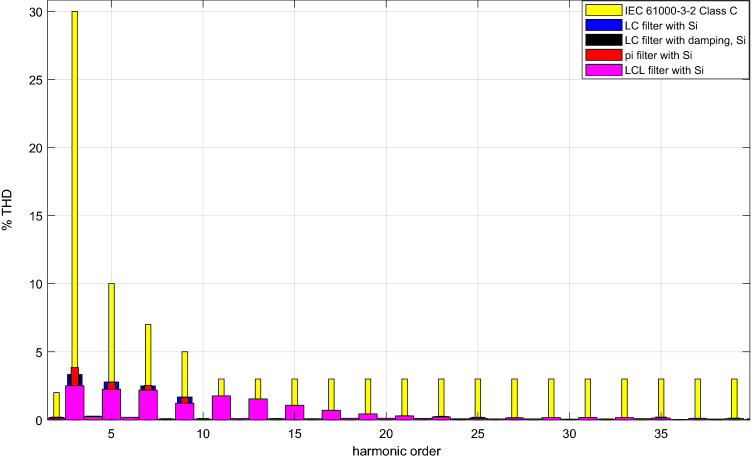
Grid current THD comparison with Si Mosfet.

By using SiC Mosfet with each of the DM filter topologies, grid current THD’s are shown in Fig. 36. Also, in the same figure comparison is made with C class of IEC61000-3–2 standard. In addition, the same comparison is realized by using Si Mosfet in Fig. 37. As a result, both power switches ensure the IEC61000-3-2 C class limit with each DM filter. Also, they provide B and A classes.

**Figure 38:**
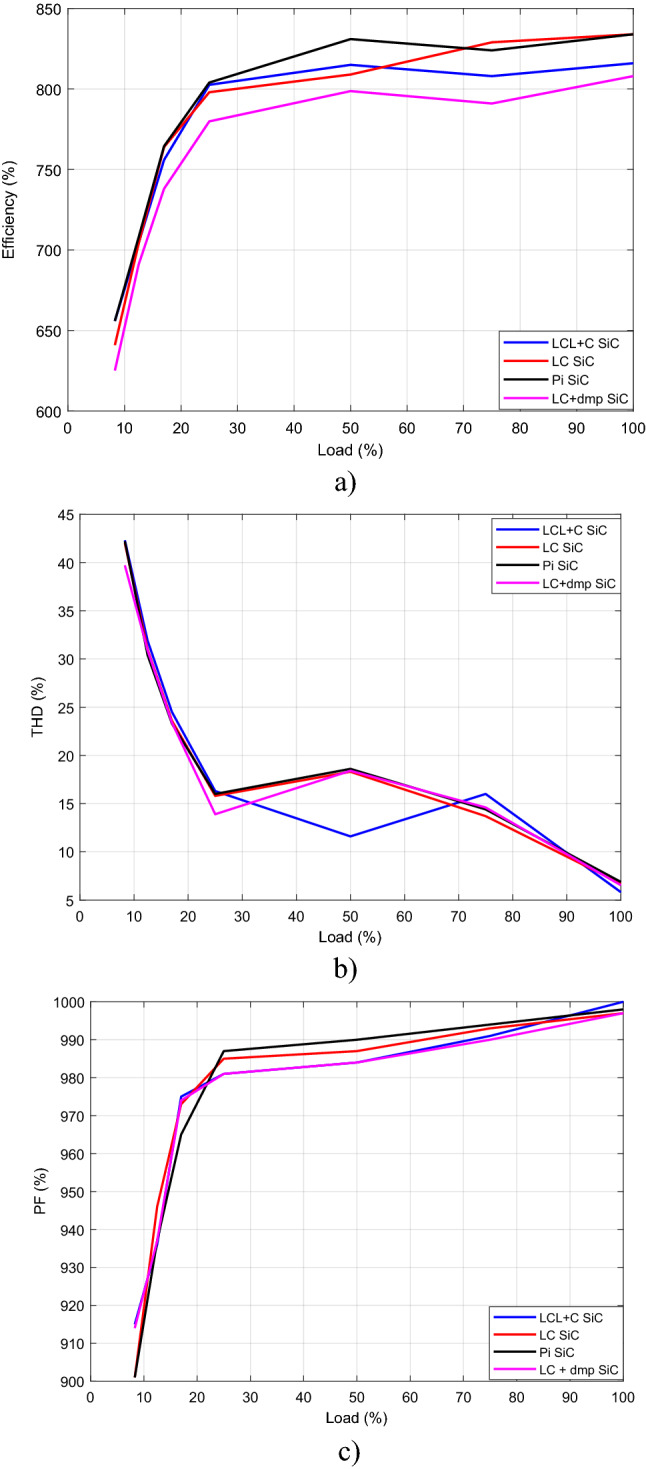
(**a**) Efficiency, (**b**) THD, (**c**) PF for SiC Mosfet by load change.

Efficiency, THD and PF graph by load change for SiC mosfet is sketched in Fig. 38, after measurements. It is seen by Fig. 38 that, the best efficiency, the best THD and the best PF are obtained at full load, 12.5 Ω.

**Figure 39:**
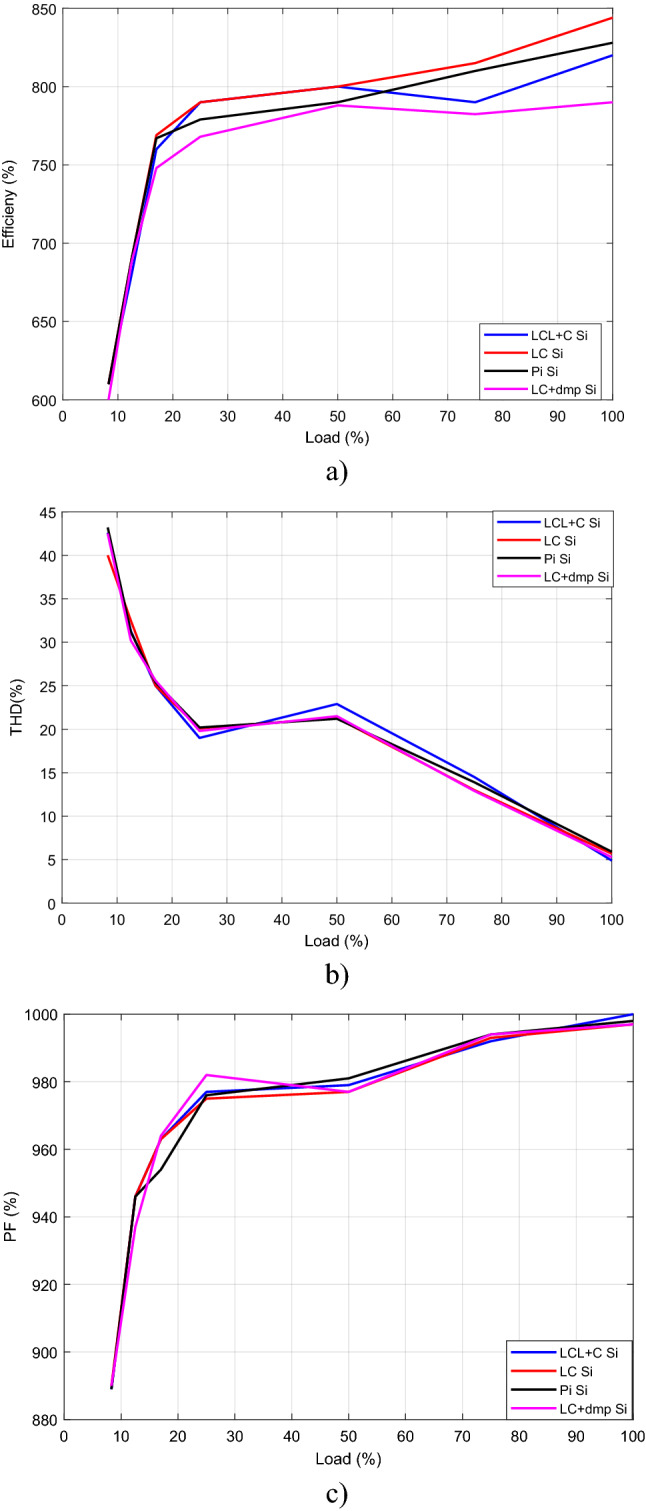
(**a**) Efficiency, (**b**) THD, (**c**) PF for Si Mosfet by load change.

Efficiency, THD and PF graph by load change for Si mosfet is sketched in Fig. 39, after measurements. It is seen that the best efficiency, the best THD, the best PF are obtained at full load, 12.5 Ω.

It is also observed by the Fig. 33 that diode losses are the highest losses. In the application, FESF16JT 600 V, 16 A diode is employed having 1.5 V forward voltage drop. So, changing it, with another lower voltage drop diode such as Schottky diode will provide by 3–5% efficiency increase.

Comparison regarding to the filter structure is summarized in Table 2. The comparison includes component counts, key results in terms of THD, PF and efficiency.

**Table 2:** Filter comparison.

	Component count	Current THD (%)	PF	η (%)	Contr. gain
LC	2	5.69	0.997	83	3.089 × 10^–4^
LC with dmp.	4	5.29	0.997	80	0.0576
Pi	3	5.91	0.998	83	3.089 × 10^–4^
LCL + C	5	4.9	1	82	0.7585

Although the number of components of LCL with C filter is higher than the other filter topology, LCL + C filter ensures the advantages of reducing total inductor value by 45%.

In Table 3, isolated PFC Ćuk converter topologies in^42–45^ and the presented paper are compared with respect to efficiency, operating mode of inductors, PF, THD, power, and filter types. It is concluded by the Table 3 that efficiency of presented paper is high enough. Presented paper is for 50W power, but its efficiency is close to the efficiencies obtained in the applications for more than 250 W to 3.3 kW. Also, THD and PF conducted in this paper are similar or better than the applications presented in the literature.

**Table 3:** Comparison of presented paper with the application in literature.

Ref	Efficiency (%)	THD (%)	PF	L_1_	L_2_	Filter type	Power	Phase
^42^	85 at full, 70–75 at 50W	3.7–7.5	0.97–1	CCM	DCM	LC	250 W	1
^43^	95 at full, 91 at 500W	4.41–4.87	0.999	CCM	DCM	L	1.6 kW	3
^44^	91.5 at full	7.24	0.997	CCM	CCM	–	3.3 kW	3
^45^	80 at full	6.49	0.998	DCM	CCM	Pi	50 W	1
Presen. paper	83 at full	4.9–6.88	0.97–1	DCM	CCM	LC, Pi, LC with dmp, LCL + C	50 W	1

## Conclusions

In this paper, designs, models, and transfer functions of DM filter topologies, consisting of pi, LC, LC with damping LCL filters, are presented. In addition, each of the DM filters are applied by PFC isolated Ćuk converter operated at DCM up to 50 W power and comparisons are made. Although, the usage of LCL filter for inverter is so popular, LCL filter application is not included in literature in detail for single phase PFC converters as it is presented in the paper as a contribution. Moreover, THD of grid current, PF and efficiency are compared for PFC isolated Ćuk converter by employing SiC and Si Mosfet independently, as another contribution of the paper such comparison is not made in literature.

Besides, average state-space model derivation for DCM operation for input side inductor of isolated PFC Ćuk converter is realized that is not introduced in literature. It is the main contribution of the paper. Also, cascaded transfer functions analyses of DM filters and the converter through bode and root locus which are linear control techniques for PFC converter is unique contribution of the study. Owing to the linear methods, stability analysis of each filter has been accomplished. Correctness of the analysis and the transfer functions are validated by step response graph. Also, it is shown by applications and step responses that same output voltage is obtained by lower duty cycle of LCL with parallel C DM filter than other DM filters. As a result, with respect to the maximum gain, LCL with C filter having 0.7585 is better than other types of filters. Furthermore, output voltage regulation characteristics of each filter is similar.

Thanks to the application, it is observed that LCL filter solely does not give proper results because it changes the operation mode of the converter. So, presented LCL with parallel C DM filter provides better outcomes by 4.9% of current THD and PF as ‘1’. In addition, LCL with parallel C filter ensures 45% total inductor reduction comparing to LC derived types of DM filters. Moreover, each DM filter ensures higher PF and lower THD that are at least 0.997 and 6.88% respectively. Furthermore, because of the damping resistor, lower efficiency as 80% is obtained by LC with damping DM filter. Also, similar efficiencies are obtained by LC, pi and LCL with parallel C DM filters as 83%, 83% and 82%, respectively. Besides, power loss calculation of each element and efficiency graphs by load percentage support the application results of efficiency.

After power switch comparison by applications, it is concluded that the best efficiency value is conducted by SiC Mosfet as 83% and best current THD is conducted by 4.9% by Si Mosfet. It is also shown by application results that all DM filters with each power switches ensures the IEC6100-3-2 standard. Moreover, the best efficiency is obtained as 83% which is ideal for low power application that is 50 W converter. Further, the efficiency is compared with the same converter topology at different power level in the literature. It is also validated by the comparison that 83% efficiency is even better than some application in the literature. However, by changing power diode with the diode having low voltage drop, it is assumed that 2% efficiency increase will be obtained.

As a future work, comparison of the filter types will be realized by using GaN power switch.

## Data Availability

All data generated or analysed during this study are included in this published article.
